# Protocol to decellularize porcine right ventricular outflow tracts using a 3D printed flow chamber

**DOI:** 10.1016/j.xpro.2024.102899

**Published:** 2024-02-16

**Authors:** Amy G. Harris, Vico Schot, Michele Carrabba, Dominga Iacobazzi, Mohamed T. Ghorbel, James P.K. Armstrong, Adam W. Perriman, Massimo Caputo, Giovanni Biglino, Francesca Bartoli-Leonard

**Affiliations:** 1 Bristol Heart Institute, Bristol Medical School, University of Bristol, BS2 8HW Bristol, UK; 2 Department of Translational Health Sciences, Bristol Medical School, University of Bristol, BS1 3NY Bristol, UK; 3 School of Cellular and Molecular Medicine, Biomedical Sciences Building, University Walk, BS8 1TD Bristol, UK; 4 Cardiac Surgery, University Hospitals Bristol, NHS Foundation Trust, BS2 8HW Bristol, UK; 5 Cardiorespiratory Unit, Great Ormond Street Hospital for Children, NHS Foundation Trust, WC1N 3JH London, UK

**Keywords:** Immunology, Tissue Engineering

## Abstract

Surgical treatment of pediatric congenital heart disease with tissue grafts is a lifesaving intervention. Decellularization to reduce immunogenicity of tissue grafts is an increasingly popular alternative to glutaraldehyde fixation. Here, we present a protocol to decellularize porcine right ventricular outflow tracts using a 3D printed flow chamber. We describe steps for 3D printing the flow rig, preparing porcine tissue, and using the flow rig to utilize shear forces for decellularization. We then detail procedures for characterizing the acellular scaffold.

For complete details on the use and execution of this protocol, please refer to Vafaee et al.^1^

## Highlights

• Successful *in vitro* system to decellularize porcine right ventricular outflow tracts
• Three-dimensional printing of a chamber to facilitate continuous fluid flow
• Simultaneous decellularization of the pulmonary artery, valve, and right ventricle
• Characterization of the acellular scaffold for cell removal and structural integrity

## Abstract (editor highlights)

Publisher’s note: Undertaking any experimental protocol requires adherence to local institutional guidelines for laboratory safety and ethics.

## Graphical abstract

**Figure:**
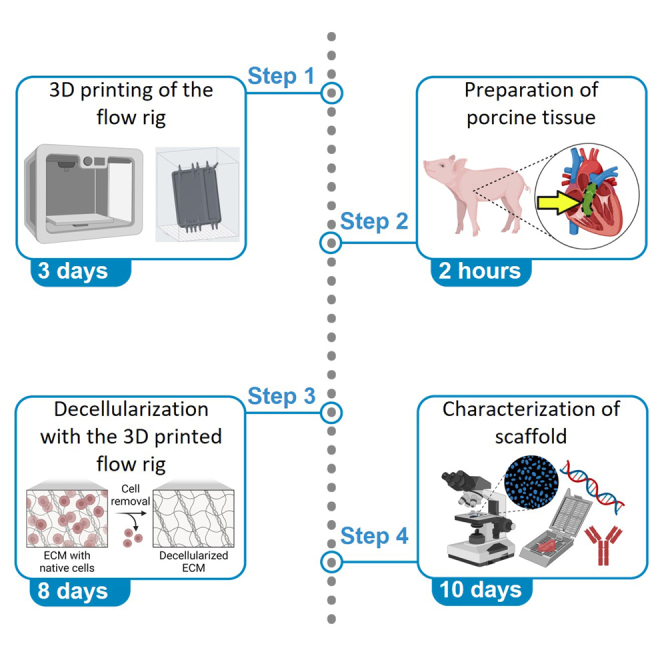

## Before you begin

The protocol describes an optimized step-by-step methodology employed to decellularize porcine right ventricular outflow tracts (RVOTs) of various sizes, as well as the characterization steps required for process quality control. This process produces acellular scaffolds by simultaneously employing three techniques, namely continuous fluid flow, enzymatic, and chemical decellularization. This protocol was successfully used to achieve cell and immunogen removal in combination with preservation of the underlying extracellular matrix (ECM) structural integrity. We have used this method to decellularize both fresh and frozen RVOTs, and for the latter we outline a freezing step that has no detrimental effect on the protocol success. This is important for clinical translation as it enables flexibility in sample acquisition, for example, from tissue banks with long-term frozen samples.

### Institutional permissions

Porcine RVOT valved conduits were obtained, as waste tissue, from companion and blood donor female Landrace pigs (20–100 kg) covered by UK Home Office Project License PP0950206. Animals were treated in accordance with the “Guide for the Care and Use of Laboratory Animals” published by the National Institutes of Health in 1996 and conforming to the “Animals (Scientific Procedures) Act” published in 1986.

It is essential to acquire all legal and ethical permissions from the relevant institutions prior to conducting this work.

### Preparation of solutions

**Timing: 2 h, variable**

#### Decellularization solutions

The volume of each decellularization stock solution is dependent on the volume of the decellularization chamber. For example, in the 250 mL chamber fabricated using this protocol (available at GitHub: 3D flow rig for decellularization), the following volumes of stock solutions are required for effective decellularization:

3 L phosphate buffered saline (PBS).

  Purpose: thorough washing to remove debris and remnants of decellularization solutions.

RVOT decellularization solution 1; 500 mL 10 mM Tris.

  Purpose: hypotonic buffer to cause water to enter cells and aid in membrane rupture.

RVOT decellularization solution 2; 2.5 mL 10% (w/v) sodium dodecyl sulphate (SDS).

  Purpose: detergent that solubilizes cell membranes, disrupting protein-lipid and protein-protein interactions.

RVOT decellularization solution 3; 500 mL 50 mM Tris + 1 mM magnesium chloride (MgCl_2_).

  Purpose: hypertonic buffer to cause water to exit cells and lyse. Magnesium acts as the buffer for the nuclease Benzonase, which is later added to the working solution for cleavage of the phosphodiester bonds between nucleotides of nucleic acids.

RVOT decellularization solution 4; 250 mL 50 mM Tris + 1.5 M sodium chloride (NaCl).

  Purpose: hyperosmotic solution to cause further cell shrinkage and lysis.

Below are example volumes for each stock solution and can be scaled according to the chamber volume. Solutions should be thoroughly mixed on a magnetic stirrer until all components are fully dissolved. The decellularization stock solutions must be prepared in advance, prior to setting up the decellularization apparatus. In part 3d: decellularization procedure, these stocks are combined with other reagents to produce the working solutions for decellularization immediately before use.

1. PBS.
  a. Dissolve 5 PBS tablets in 1 L distilled (d)H_2_O.
  b. Autoclave (121°C for 15 min).
  c. Store at room temperature (usually 18°C–22°C) for up to 6 months.
2. RVOT decellularization solution 1 (10 mM Tris).
  a. Dissolve 1.21 g Trizma base (molecular weight 121.14 g/mol) in 950 mL dH_2_O.
  b. Thoroughly mix and adjust pH to 8.0 by adding 1 M sodium hydroxide (NaOH) if pH is under 8.0 or 37% (w/w) hydrochloric acid (HCl) if pH is above 8.0.
  c. Make to 1 L with dH_2_O using a 1 L volumetric flask for high accuracy measurement.
  d. Autoclave (121°C for 15 min).
  e. Store at room temperature (usually 18°C–22°C) for up to 6 months.
3. RVOT decellularization solution 2 (10% (w/v) SDS stock).
  a. Dissolve 1 g SDS in 10 mL dH_2_O.
  b. Store at room temperature (usually 18°C–22°C) for up to 6 months.
4. RVOT decellularization solution 3 (50 mM Tris + 1 mM MgCl_2_).
  a. Dissolve 6.05 g Trizma base (molecular weight 121.14 g/mol) and 0.10 g MgCl_2_ (molecular weight 95.21 g/mol) in 950 mL dH_2_O.
  b. Thoroughly mix and adjust pH to 8.0 by adding 1 M NaOH if pH is under 8.0 or 37% (w/w) HCl if pH is above 8.0.
  c. Make to 1 L with dH_2_O using a 1 L volumetric flask for high accuracy measurement.
  d. Autoclave (121°C for 15 min).
  e. Store at room temperature (usually 18°C–22°C) for up to 6 months.
5. RVOT decellularization solution 4 (50 mM Tris + 1.5 M NaCl).
  a. Dissolve 6.05 g Trizma base (molecular weight 121.14 g/mol) and 87.66 g NaCl (molecular weight 58.44 g/mol) in 950 mL dH_2_O.
  b. Thoroughly mix and adjust pH to 8.0 by adding 1 M NaOH if pH is under 8.0 or 37% (w/w) HCl if pH is above 8.0.
  c. Make to 1 L with dH_2_O using a 1 L volumetric flask for high accuracy measurement.
  d. Autoclave (121°C for 15 min).
  e. Store at room temperature (usually 18°C–22°C) for up to 6 months.

#### Characterization solutions

6. 10 mM citrate buffer for immunohistochemistry (IHC).
  a. Dissolve 1.92 g citric acid (molecular weight 192.12 g/mol) in 950 mL dH_2_O.
  b. Thoroughly mix and adjust pH to 6.0 by adding 1 M NaOH if pH is under 6.0 or 37% (w/w) HCl if pH is above 6.0.
  c. Make to 1 L with dH_2_O using a 1 L volumetric flask for high accuracy measurement.
  d. Store at 4°C for up to 3 months.
7. 0.2 M sodium cacodylate in 5% (v/v) glutaraldehyde for scanning electron microscopy (SEM) fixation.
  a. Make 0.2 M sodium cacodylate stock: dissolve 2.14 g sodium cacodylate (molecular weight 214.03 g/mol) in 50 mL dH_2_O.
  b. Store at 4°C for up to 6 months.
  c. Make 5% (v/v) glutaraldehyde stock: dilute 10 mL 25% (v/v) glutaraldehyde to 5% (w/v) by adding 40 mL dH_2_O.
  d. Store at room temperature (usually 18°C–22°C) for up to 6 months.
  e. Mix equal volumes of the above 0.2 M sodium cacodylate stock and 5% (v/v) glutaraldehyde stock.
  f. Always prepare working solution freshly.
8. 4% (w/v) formaldehyde for histology/IHC fixation.
  a. Dissolve 5 PBS tablets in 1 L dH_2_O.
  b. Working in a ventilation hood, gently add 40 g of paraformaldehyde (PFA) powder (molecular weight 30.03 g/mol) into 500 mL 1× PBS made in 8a.
  c. Increase the pH with 1 M NaOH at 60°C in a ventilation hood until the particulate dissolve, being careful not to boil.
  d. Allow the solution to cool to room temperature (usually 18°C–22°C) in a ventilation hood.
  e. In a ventilation hood, make up to 950 mL with 1× PBS.
  f. Adjust pH to 7.4 by adding 1 M NaOH if pH is under 7.4 or 37% (w/w) HCl if pH is above 7.4.
  g. Filter the solution through a 0.45 μm membrane filter to remove any undissolved PFA particles.
  h. Make to 1 L with 1× PBS using a 1 L volumetric flask for high accuracy measurement.
  i. Store at 4°C for up to 2 weeks, or store 5 mL aliquots at −20°C for up to 1 year, avoiding repeated freeze-thaw cycles.

**CRITICAL:** Handle PFA very carefully as it is a hazardous reagent. Follow the manufacturer’s guidance on how to handle and store PFA. Wear personal protection equipment (lab coat, gloves, and eye protection).

***Note:*** 4% (w/v) formaldehyde can also be made from a purchased stock solution, for example 10% (w/v) neutral buffered formalin from Fisher Scientific Ltd, cat# 10463750.

## Key resources table

**Table:** 

REAGENT or RESOURCE	SOURCE	IDENTIFIER
**Antibodies**		

α-Gal epitope (Galα1-3Galβ1-4GlcNAc-R) monoclonal antibody M86. Used at 1:5 dilution	Enzo Life Sciences (UK) Ltd.	Cat# ALX-801-090-1
Biotin-labeled goat polyclonal anti-mouse IgM. Used at 1:250 dilution	Cambridge Bioscience Ltd.	Cat# A90-101B

**Biological samples**		

Porcine right ventricular outflow tracts	Translational Biomedical Research Centre (TBRC) at the Veterinary School of the University of Bristol, UK	N/A

**Chemicals, peptides, and recombinant proteins**		

3,3′-Diaminobenzidine	Abcam	Cat# ab64238
AE buffer	QIAGEN	Cat# 19077
Benzonase nuclease	Merck Life Science UK Ltd.	Cat# 70746-3
BLOXALL	BioServ UK Ltd.	Cat# VEC-SP-6000
Citric acid	Sigma-Aldrich	Cat# C0759-500G
Clearing agent	Merck Life Science UK Ltd.	Cat# H2779-1L
DPX mountant	Sigma-Aldrich	Cat# 317616-100ML
D-limonene	Magnacol Ltd.	Limonene
Eosin yellowish	Alfa Aesar	Cat# 17372-87-1
Ethanol	Sigma-Aldrich	Cat# 32205-2.5L-M
Ethylenediaminetetraacetic acid	Millipore Corp.	Cat# 324506-100ML
Extravidin-horseradish peroxidase	Merck Life Science UK Ltd.	Cat# E2886-1ML
Glutaraldehyde	Sigma-Aldrich	Cat# G6257-100ML
Hanks’ balanced salt solution	Fisher Scientific Ltd.	Cat# 15266355
Hydrochloric acid	Fisher Scientific Ltd.	Cat# 10294190
Magnesium chloride	Fisher Scientific Ltd.	Cat# 10697473
Mayer hematoxylin	VWR International	Cat# 10047005
Millers elastin stain	Pioneer Research Chemicals Ltd.	Cat# PRC/R/108
Milton	VWR International	Cat# DIUKMIL0012
NE buffer set (r1.1 r2.1 r3.1 and rCutSmart)	New England Biolabs Ltd.	Cat# B7030S
Normal horse serum	Merck Life Science UK Ltd.	Cat# H0146-10ML
Nuclease P1	New England Biolabs Ltd.	Cat# M0660S
Oxalic acid	Sigma-Aldrich	Cat# O0376-500G
Paraformaldehyde	Sigma-Aldrich	Cat# P6148-500G
Penicillin/streptomycin	Life Technologies	Cat# 15140-122
Phosphate-buffered saline tablets	Scientific Laboratory Supplies Ltd.	Cat# P4417-100TAB
Pierce protease inhibitor tablets EDTA-free	Thermo Fisher Scientific	Cat# A32965
Potassium permanganate	VWR International	Cat# 3227-01
ProLong gold antifade mountant with DAPI	Life Technologies	Cat# P36941
Propan-2-ol	Sigma-Aldrich	Cat# 59300-2.5L
Scott’s tap water substitute	Merck Life Science UK Ltd.	Cat# S5134-6X100ML
Sodium cacodylate	Sigma-Aldrich	Cat# 20840-25G-F
Sodium chloride	Fisher Scientific Ltd.	Cat# 10728214
Sodium dodecyl sulfate	Sigma-Aldrich	Cat# 1001158542
Sodium hydroxide	Fisher Scientific Ltd.	Cat# 10528240
Trizma base	Sigma-Aldrich	Cat# 93362
UltraPure DNase/RNase-free distilled water	Life Technologies Ltd.	Cat# 10977035
Van Gieson stain	Pioneer Research Chemicals Ltd.	Cat# PRC/R/54

**Critical commercial assays**		

DNeasy blood and tissue kit	QIAGEN	Cat# 69504
Invitrogen Quant-iT PicoGreen dsDNA assay kit	Fisher Scientific UK Ltd.	Cat# P11496

**Software and algorithms**		

Bluehill 3	Instron	Bluehill 3
Fiji version 1.54.f	Fiji	Fiji
Formlabs software: PreForm version 3.31.0	Formlabs	PreForm
Fusion 360 (2.0.16976)	Autodesk	Fusion 360
GraphPad Prism version 6.07	GraphPad	GraphPad Prism
OlyVIA version 4.1	Olympus	OlyVIA
ZEN Blue software	ZEISS	Zen Blue

**Other**		

0.2 μm membrane filters	Sarstedt	Cat# 83.1826.001
0.45 μm membrane filters	Sarstedt	Cat# 83.1826
1 L volumetric flask	Merck Life Science Ltd.	Cat# CLS5641P1L-3EA
3-way stopcock	Fisher Scientific UK Ltd.	Cat# 17534546
3D printer, firmware version RC-2.0.0-825 (2019)	Formlabs	Model: Form 3
50 mL tubes	Sarstedt	Cat# 62.559.001
50 mL syringes	Appleton Woods	Cat# BD588
96-well black plates with lid	Greiner Bio-One Ltd.	Cat# 655086
Adhesive carbon tabs	Agar Scientific	Cat# AGG3347N
Autoclave	Astell Scientific Ltd.	Model: AMB430BT65
Autostainer	Thermo Scientific	Model: A81500001
Benchtop centrifuge	Fisher Scientific UK Ltd.	Cat# 75002460
BioMmed clear resin	Formlabs	Cat# RS-F2-BMCL-01
Blunt-end narrow forceps	Fisher Scientific UK Ltd.	Cat# 12391369
Blunt-end surgical scissor	Fisher Scientific UK Ltd.	Cat# 12368099
Cartridge pump	Masterflex, Cole-Parmer	Model: 07528-10
Cartridge pump head	Masterflex, Cole-Parmer	Model: 7519-06
Class II microbiological safety cabinet	NuAire	Model: NU-543-4005
Cole-Parmer Masterflex peroxide-cured silicone L/S precision pump tubing, tubing size: 16	Fisher Scientific UK Ltd.	Cat# 11795138
Critical point dryer	Leica Microsystems	Model: CPD300
CryoPure 1.6 mL tubes	Sarstedt	Cat# 72.380.002
Digital section bath	Thermo Scientific	Model: SB1661D1509
Elastic 50A resin	Formlabs	Cat# RS-F2-ELCL-01
Filling tubes	Sarstedt	Cat# 94.6077.138
Form 3 resin tank V2.1	Formlabs	Cat# RT-F3-02-01
Form Cure (2019)	Formlabs	Model: Form Cure
Form Wash (2018)	Formlabs	Model: Form Wash
Freeze dryer	Edwards	Model: Modulyo
GloMax Discover	Promega	Model: 9700000322
G-clamps	Toolstation	Cat# 86676
Heat block	Jencons-PLS	Model: D1200-230V
Histology cassettes	VWR International	Cat# 720-0887
Hose barb, 1/8″ male luer (for tubing)	Ezkem	Cat# A002797
Hose barb, 3/16″ male luer (for tissue)	Cole-Parmer	Cat# WZ-12028-52
ImmEdge hydrophobic barrier pen	Bio-Techne	Cat# 310018
Impulse sealer	TEW	Model: E82163(S) TISH-300
Incubator	Heraeus Instruments	Model: B 6060
Instron tensile tester	Instron	Model: 3343
Micrometer	Onecall	Cat# 3377020
Microscope cover glasses	VWR International Ltd.	Cat# ECN631-1574
Microslide SuperFrost Plus	VWR International Ltd.	Cat# 631-0108
Microtome	Thermo Fisher Scientific	Model: Shandon FINESSE 325
Microwave	Sharp	Model: R-246
Milton	VWR International	Cat# MIL0012
Petri dish	Greiner Bio-One	Cat# 664160
pH meter	Mettler Toledo	Model: FiveEasy
Polythene self-seal bags 125 x 190 mm	Appleton Woods	Cat# BW128
Polythene self-seal bags 400 x 450 mm	Fisher Scientific Ltd.	Cat# 12894128
Pump cassette	Masterflex, Cole-Palmer	Model: 07519-70
Razor blades	Rapid Electronics Ltd.	Cat# 96-7757
Reaction tubes	Greiner Bio-One	Cat# 616201
Scalpel	Fisher Scientific Ltd.	Cat# 0509
Scanning electron microscope	FEI	Model: Quanta 200 F
Scanning electron microscopy specimen stubs	Agar Scientific	Cat# AGG301
Slide scanner	Olympus	Model: VS200
Sputter coater	Emitech	Model: K575X
Staining rack	VWR International	Cat# 631-0348
Stirrer	Jencons-PLS	Model: KM02 BS2
Surgical straight scissors	Fisher Scientific UK Ltd.	Cat# 12912055
Sutures 4-0	Ethicon	Cat# W8683
Tissue block system cool unit	MEDITE	Model: TB588.4
Tissue embedder	Thermo Scientific	Model: HistoStar
Tissue processor	Thermo Scientific	Model: Excelsior AS
TissueVault cryogenic freezing bags 9 × 18 cm	OriGen Biomedical Inc.	Cat# TV0918
TissueVault cryogenic freezing bags 9 × 9 cm	OriGen Biomedical Inc.	Cat# TV0909
Vernier calipers	Rapid Electronics Ltd.	Cat# PGA1001
Water bath	Grant Instruments Ltd.	Model: UE1821006 SAP12
ZEISS Axio Observer.Z1	ZEISS	Model: 1026044910

## Step-by-step method details

### Part 1: Preparation of the 3D printed flow rig

**Timing: total printing time, 43 h 15 min; total wash time, 1 h; total curing time, 2 h 20 min**

**Figure 1:**
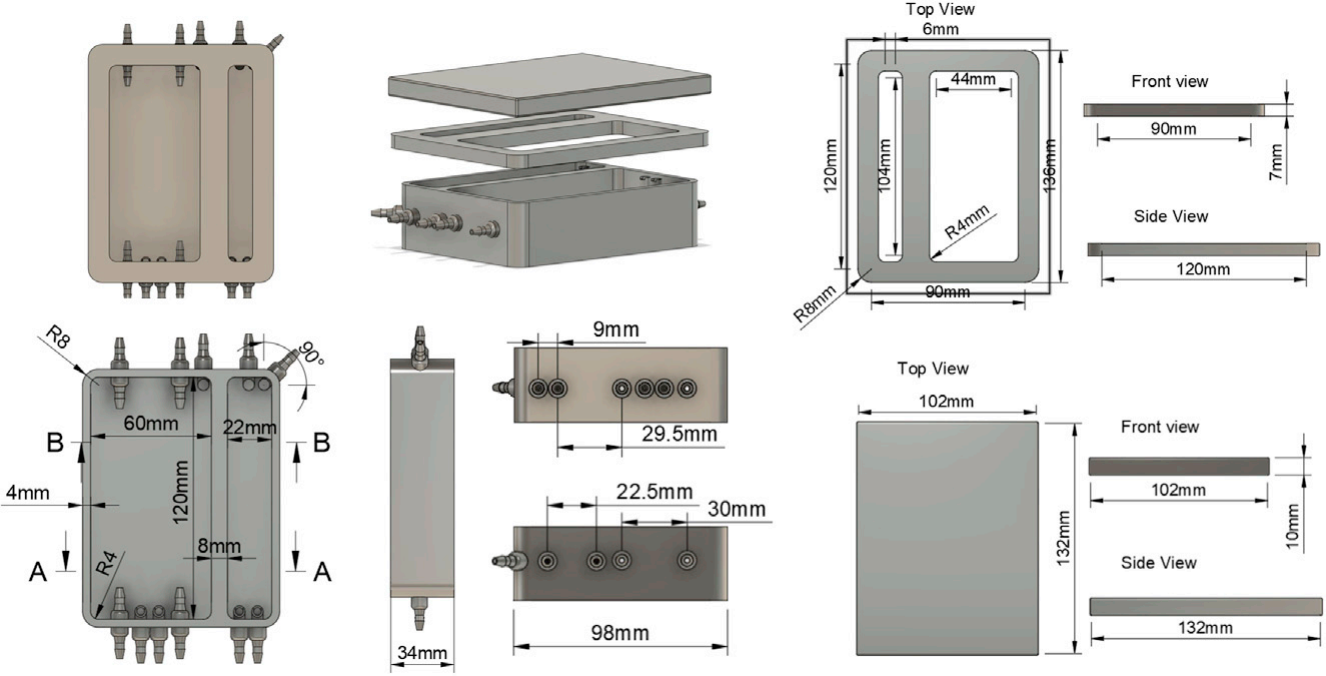
Schematic of the 3D printed flow chamber, gasket, and seal with dimensions indicated

In the first step, the flow rig necessary for efficient decellularization must be 3D printed (Figure 1). The flow rig is the apparatus in which the RVOTs are decellularized and comprises the bioreactor chamber with an integrated reservoir, gasket, and chamber lid. Together, these components provide a closed environment wherein wash and decellularization solutions are circulated over the 8-day period.

***Note:*** Additional flexibility can be achieved as users can experiment with their own 3D printing equipment and produce similar parts, ensuring the biocompatibility of the utilized resins.

***Note:*** The linked downloadable 3D printed flow rig (available at GitHub: 3D flow rig for decellularization) utilizes commercially available resins that are USP Class VI certified materials made in an FDA-registered, EU MDR and ISO 13485 certified facility, namely BioMed Clear Resin RS-F2-BMCL-01 (chamber and lid) and Elastic 50A Resin RS-F2-ELCL-01 (gasket).

***Note:*** Equivalent flow rigs can be produced on alternative 3D printers, including the Form 2, 3+, 3B, 3B+, 3L, and 3BL.

***Note:*** The flow rig accommodates RVOTs of different sizes and allows the decellularization process to be parallelized, decellularizing two RVOTs simultaneously. We have tested RVOTs of up to 22 mm diameter, obtained from approximately 20–100 kg pigs, with no apparent change in efficacy of decellularization as sample diameter increases, demonstrated in part 4: characterization of decellularized tissue.

***Note:*** Though we recommend the above resins, alternative resins can be used if necessary. The required key features of the resins are their ability to be sterilized as well as being cell culture compatible. The material for the gasket must be appropriately soft to enable a full seal between the chamber and lid.

**CRITICAL:** Check the correct curing times for all resins if changing from those suggested here. Curing for too long will produce a brittle part, whereas insufficient cure time will result in an overly soft product.

***Note:*** BioMed Clear Resin and Elastic 50A Resin have been validated for five rounds of sterilization by Formlabs, where 3D printed test samples underwent five rounds of pre-vacuum steam sterilization at 132°C involving a four minute sterilization phase and 30 minute dry phase, with 30 minutes between cycles. Material property losses, cracking, deformations, nor color changes were observed.

**Table:** 

	Chamber	Lid	Gasket
Printer	Form 3	Form 3	Form 3
Print time	16 h 15 min	9 h 45 min	17 h 15 min
Resin	RS-F2-BMCL-01	RS-F2-BMCL-01	RS-F2-ELCL-01
Support touchpoint size	0.5 mm	0.5 mm	0.6 mm
Support touchpoint density	1.0 kg/m^3^	1.0 kg/m^3^	0.65 kg/m^3^
Washing	Flush all inlets with propan-2-ol using a 50 mL syringe, ensuring all excess resin is removed from these structures. Remove supports with clippers and tweezers, being careful not to damage the chamber. Wash with the Form Wash, submerging and agitating the chamber in propan-2-ol for 20 min.	Rinse with propan-2-ol. Remove supports with clippers and tweezers, being careful not to damage the lid. Wash with the Form Wash, submerging and agitating the lid in propan-2-ol for 20 min.	Rinse with propan-2-ol. Remove supports with clippers and tweezers, being careful not to damage the gasket. Wash with the Form Wash, submerging and agitating the gasket in propan-2-ol for 20 min.
Curing temperature	60°C	60°C	60°C
Curing time	60 min	60 min	20 min
Autoclave	121°C for 15 min	121°C for 15 min	121°C for 15 min
.stl file	Chamber	Lid	Gasket

**CRITICAL:** When cleaning the parts following printing, thoroughly flush all inlets and outlets with a 50 mL syringe and propan-2-ol to prevent blockage by uncured resin.

**CRITICAL:** Once cured, autoclave the chamber, lid, and gasket at 121°C for 15 min to ensure sterility prior to use.

### Part 2: Preparation of tissue: Dissecting, washing, and freezing porcine RVOTs

**Timing: dissection: 15 min per heart, variable**

**Timing: washing and freezing: 1 h, variable (for step 10-21)**

The heart is first dissected to separate the RVOT for decellularization. Following this, the RVOT is extensively washed to remove debris and frozen at −80°C to preserve the tissue until use.

**CRITICAL:** The porcine hearts should be collected as soon as possible following explant to prevent infection and deterioration.

***Note:*** Here, tissue was collected on the day of explant as waste tissue from an animal research unit, however sourcing tissue as a byproduct of the food industry can be an alternative supply **(**troubleshooting, problem 1**).**

**CRITICAL:** The dissection is carried out in clean but not sterile conditions, given that the tissue was not obtained from a sterile source. However, all further wash steps should be conducted aseptically in a class II microbiological safety cabinet. The use of autoclaved (121°C for 15 min) or dry heat sterilized glassware, forceps, and scissors is necessary to avoid handling-associated microbial contamination. Additionally, the PBS wash solution should be autoclaved (121°C for 15 min) before use.

**Figure 2:**
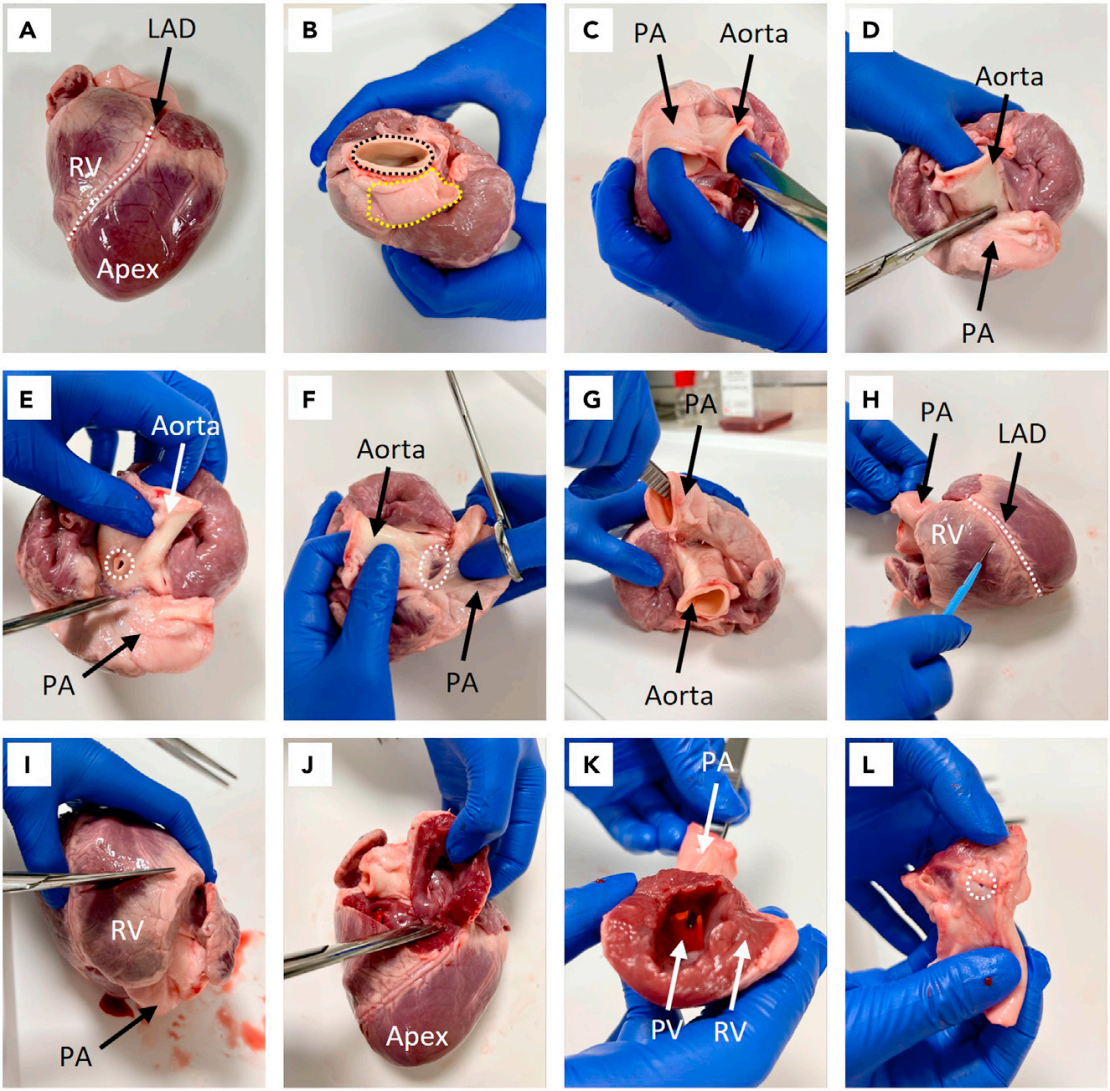
Dissection of a porcine heart (A) Porcine heart with the apex facing the operator and the right ventricle (RV) orientated at the top. The left anterior descending (LAD) artery is marked in white. (B) Identification of the pulmonary artery, marked in yellow, and the aorta, marked in black. (C and D) Separation of the pulmonary artery (PA) and aorta with blunt-end scissors. (E) Example of acceptable aortic damage, marked in white, incurred during dissection. The RVOT is undamaged. (F) Exposure of the top of the right ventricle, marked in white. (G) Insertion of blunt-end tweezers into the pulmonary artery, through the pulmonary valve. (H) Incision into the right ventricle, against the end of the tweezers. (I and J) Separation of the RVOT from the rest of the heart. (K) The final dissected RVOT, comprising of the pulmonary artery, pulmonary valve (PV), and right ventricle, is ready for washing. (L) Example of unacceptable pulmonary artery damage incurred during dissection, marked in white. This damaged RVOT should be discarded.
3. Distinguish the pulmonary artery (indicated in yellow) from the aorta (indicated in black) (Figure 2B).

***Note:*** The aorta originates from the left ventricle and is posterior to the pulmonary artery, which originates from the right ventricle. It may be necessary to remove excess blood or clots from the tissue to enable proper visual assessment.

1. Collect the porcine heart in a heavy-duty polythene bag and transport to the laboratory on ice.
2. Working on a clean surface suitable for dissection, place the heart in the orientation shown in Figure 2A.

4. Gently pull the aorta and pulmonary artery away from each other to reveal the fissure between the arteries (Figure 2C).
5. Using blunt-end scissors, cut downwards against the aorta, gently cutting the adventitia layer-by-layer (Figure 2D).

***Note:*** Damage to the aorta does not matter, and cutting against this artery prevents damage to the pulmonary artery. An example of acceptable aortic damage is shown in Figure 2E, circled in white.

6. Continue to carefully separate the arteries until reaching the top of the heart (Figure 2F).
7. Carefully insert blunt-end tweezers into the pulmonary artery, through the pulmonary valve and into the right ventricle (Figure 2G).
8. Push the tweezers against the right ventricle wall and cut the right ventricle against the end of the tweezers with a scalpel (Figure 2H).
9. Begin cutting the right ventricle with scissors, separating the RVOT from the rest of the heart (Figures 2I and 2J).

***Note:*** The final dissected RVOT is shown in Figure 2K.

10. Place the dissected RVOT in an appropriately sized container and submerge in sterile PBS with 1× penicillin/streptomycin (P/S).
11. Sterilize the outside of the container with 70% (v/v) ethanol and move to a class II microbiological safety cabinet.

**CRITICAL:** The following steps are conducted under sterile conditions, working in a class II microbiological safety cabinet.

**CRITICAL:** Carefully visually inspect the pulmonary artery for damage. Samples with even minor holes in the pulmonary artery must be discarded as integrity is compromised. An example of unacceptable pulmonary artery damage incurred during dissection is circled in white in Figure 2L. This RVOT should be discarded.

**CRITICAL:** If signs of infection such as pus are evident within any part of the heart, immediately discard the tissue. Thoroughly clean the surface and remove contaminated tools before processing further RVOTs.

**CRITICAL:** Washing of the RVOTs is essential to remove debris and blood clots that would otherwise block the apparatus. Washing also allows proper examination of the tissue for signs of damage that otherwise may be masked by blood.

12. Transfer the dissected RVOT into a new sterile container.
13. Completely submerge the RVOT in excess autoclaved PBS with 1× P/S.
14. Wash the tissue in autoclaved PBS with 1× P/S for 15 min under gentle agitation.

***Note:*** Gentle agitation can be achieved by slow manual swirling or placing the container on a rocker. If using a rocker, seal the container before removing from the hood and thoroughly spray the external surface with 70% (v/v) ethanol before returning to the sterile environment.

15. Discard the wash solution and gently remove any dislodged blood clots with blunt-end forceps.
16. Repeat steps 14–15 until there is no obvious sign of blood present and the PBS with 1× P/S runs colorless.

***Note:*** We recommend a minimum of three 15 min washes under gentle agitation, but more may be required, and tissue must be washed until clean.

**CRITICAL:** It is essential to completely submerge the RVOT in PBS with 1× P/S during the washes.

17. Place the RVOT in a TissueVault Cryogenic Freezing Bag of appropriate size.
18. Submerge the tissue in Hanks’ Balanced Salt Solution (HBSS).
19. Heat seal the Cryogenic Freezing Bag.
20. Place the Cryogenic Freezing Bag inside a polythene self-seal bag.
21. Store the RVOT at −80°C.

**Pause point:** RVOTs can be stored at −80°C before continuing. We have tested RVOT decellularization on samples frozen for up to one year. Longer storage times may be possible but have not been tested.

***Note:*** In this work, both fresh and freeze-thawed (F-T) RVOTs from Landrace pigs (20–100 kg) were decellularized to compare the impact of freezing on the tissue. Time from explant to processing (i.e., freezing (F-T RVOTs and F-T dRVOTs), fixation (fresh RVOTs), or fresh decellularization (fresh dRVOTs)) was a maximum of 10 days.

### Part 3: Decellularization of porcine RVOTs

#### Part 3a: Sterilize the apparatus

**Timing: total timing: 2.5 h, hands-on-time: 45 min**

Sterilizing the full apparatus, including the 3D printed chamber, lid, gasket, as well as all tubing and hose barbs, is a critical step before each decellularization setup and enables repeated use of the equipment.

22. Thoroughly rinse the apparatus with tap water, flushing all tubes, hose barbs, and chamber in/outlets with a 50 mL syringe.
23. Submerge and flush the apparatus with dish soap.
24. Leave to soak for 15 min.
25. Thoroughly rinse and flush the apparatus with tap water.
26. Submerge and flush the apparatus with 4% (v/v) Milton.
27. Leave to soak for 1 h.
28. Thoroughly rinse and flush the apparatus with tap water.
29. Leave to soak for 15 min.
30. Lay the apparatus out on paper towel to dry.
31. Package the apparatus for autoclave (121°C for 15 min), separating the chamber, seal, lid, tubes, and hose barbs.
32. Spray the incubator and clamps thoroughly with 70% (v/v) ethanol.

**CRITICAL:** All steps of the decellularization should be conducted as aseptically as possible. The use of sterile glassware, forceps, scissors, tubing, hose barbs, 3D printed chamber, lid, and gasket is necessary to avoid microbial contamination.

#### Part 3b: Prepare and mount the RVOT

**Timing: 2–3 h, variable**

This section describes the initial setup of the RVOT in the 3D printed chamber. At the end of this section, two thawed RVOTs will be mounted in the chamber and the apparatus is then ready to be sealed and transferred to an incubator for the duration of decellularization.

**CRITICAL:** All solutions should be autoclaved where possible, or alternatively filter sterilized with a 0.2 μm membrane filter immediately prior to use. Working solutions should be prepared in a sterile setting and P/S is added to all solutions at a working concentration of 1×.

33. preheat a water bath to 37°C and an incubator to 42°C.
34. Remove the Cryogenic Freezing Bag containing the frozen porcine RVOT from the outer polythene self-seal bag.
35. Submerge the Cryogenic Freezing Bag in a water bath at 37°C for 15–20 min, until the tissue is thawed.
36. Remove the Cryogenic Freezing Bag from the water bath and spray the outer surface with 70% (v/v) ethanol.
37. Transfer the Cryogenic Freezing Bag containing the thawed porcine RVOT to a class II microbiological safety cabinet.
38. Cut the bag open with sterile scissors, place the RVOT into an appropriately sized sterile container, and wash with autoclaved PBS with 1× P/S for 15 min under gentle agitation.
39. Place the RVOT into a sterile dish for setup.
40. Using sterile blunt-end forceps and surgical scissors, remove two ∼1 cm^2^ sections from the end of the pulmonary artery and right ventricle of the RVOT.
41. Aliquot the above tissue into four cryovials and store at −80°C, two per tissue type. These will act as biologically matched control tissue for DNA isolation and quantification.

**CRITICAL:** Always use sterile instruments to handle the tissue to prevent operator-derived contamination.

42. Gently insert a 3/16″ hose barb into the right ventricular opening.
43. Using 4-0 suture thread, secure the right ventricle onto the hose barb.
  a. Initially, tie the suture loosely, ensuring the barb is in the correct position.
  b. Tie a second suture thread, more tightly, securing the tissue to the barb. The ventricle should be sealed around the barb, and not come off when gently pulled.
  c. If necessary, fully secure the tissue with a third suture thread.

**Figure 3:**
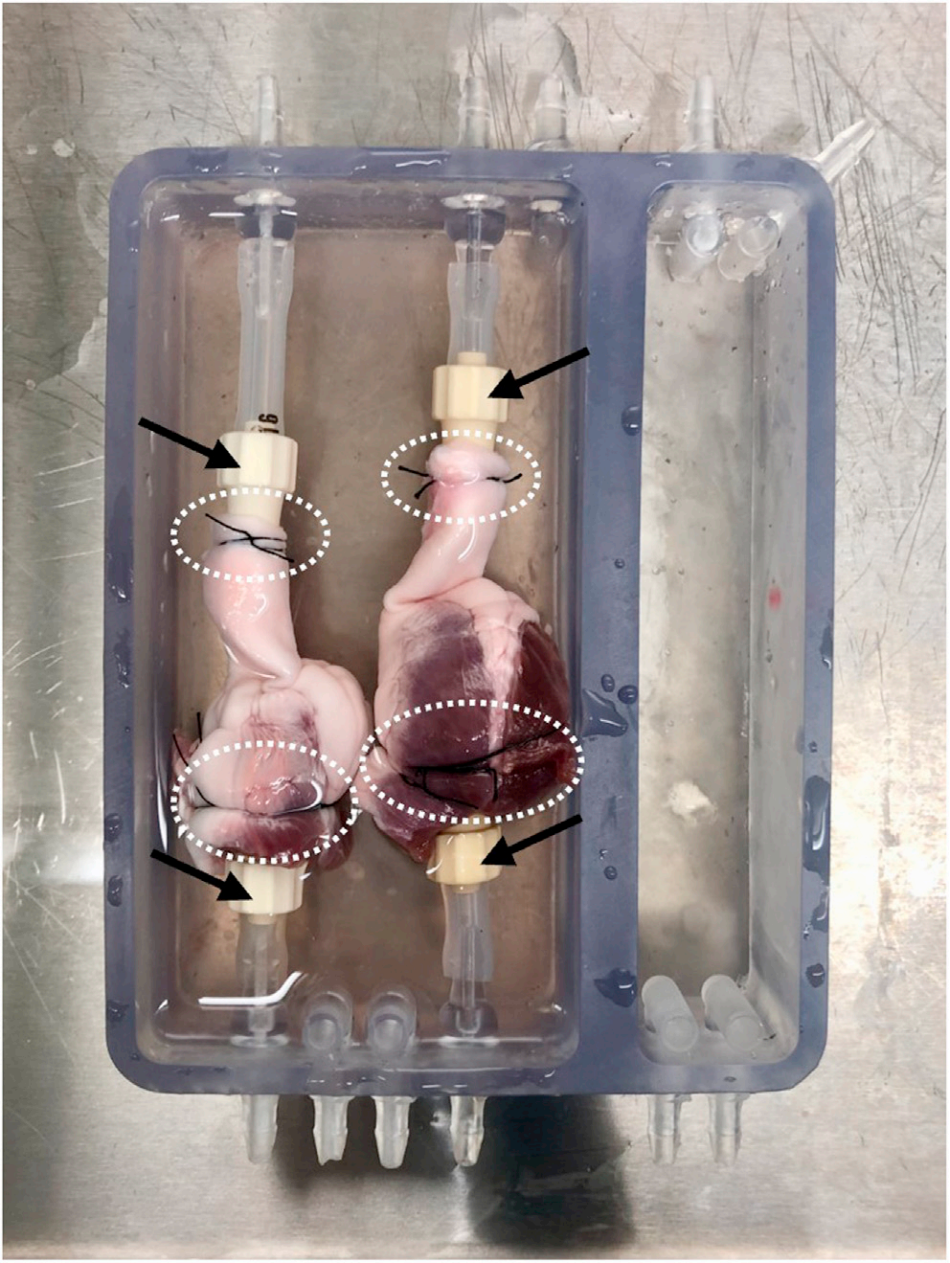
3D printed flow chamber with two mounted RVOTs Black arrows indicate the hose barbs inserted into the pulmonary artery (top) and right ventricle (bottom), with sutures marked in white.

**CRITICAL:** It is essential to not tie off the RVOT, instead place the suture above the lip of the barb (Figure 3).

44. Gently insert a second 3/16″ hose barb into the pulmonary artery opening.
45. Tie the pulmonary artery onto the hose barb, using the same method as in step 43.
46. Gently pull the hose barbs in opposite directions to ensure the RVOT does not come off.

**CRITICAL:** It is vital that the RVOT is mounted tightly onto the barbs to allow solutions to flow through the lumen of the RVOT.

47. Place the mounted RVOT into the open sample chamber (Figure 3).

**CRITICAL:** The ventricle should be at the bottom of the chamber. If mounted with the artery at the bottom, the unidirectional pulmonary valve will prevent flow through the RVOT.

48. Attach the hose barbs to the inlet with sterilized (autoclaved at 121°C for 15 min) silicone tubing (size 16), cutting this to length so the RVOT sits roughly in the middle of the chamber (Figure 3).

**CRITICAL:** The RVOT should not be stretched or compressed once attached to the chamber.

49. Add autoclaved PBS with 1× P/S to the chamber to submerge the RVOT and repeat steps 34–48 to mount the second RVOT.

***Note:*** the RVOTs are not required to be the same size/diameter for the protocol to be effective when run in parallel.

#### Part 3c: Assemble the decellularization apparatus

**Timing: 2 h, variable**

**Figure 4:**
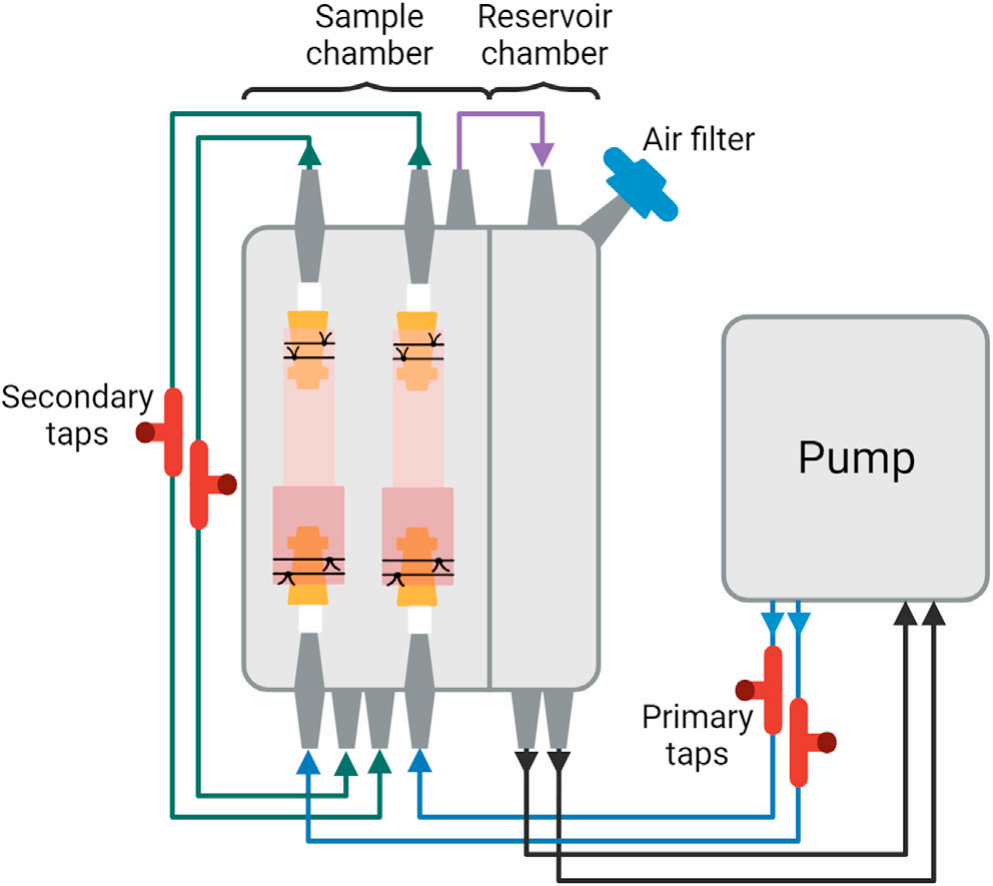
Schematic of the flow rig Blue lines indicate tubes leading from the pump into the RVOTs, green lines indicate tubes from the RVOTs to the sample chamber, the purple line indicates a tube from the sample chamber to reservoir chamber, and black lines indicate tubes leading from the reservoir chamber back to the pump. Solution should continuously flow from the pump through the primary taps and through lumen of the RVOTs, then via the secondary taps into the sample chamber, into the reservoir chamber, and finally back to the pump.

To facilitate fluid flow, the chamber is sealed with the gasket, lid, and G-clamps. The relevant tubing is then attached in a closed loop, as shown in Figure 4.

50. Place the gasket onto the chamber with the two mounted RVOTs, followed by the lid.
51. Seal the chamber with G-clamps.

**CRITICAL:** The clamps must be tightened with enough force to prevent the chamber leaking, but not overtightened as to damage the chamber.

52. Attach sterile (autoclaved at 121°C for 15 min) silicone tubing (size 16) to the chamber in/outlets via 1/8″ hose barbs to facilitate flow through the RVOTs, following the green and purple arrows in Figure 4.

***Note:*** The three-way Luer locks, referred to as the “secondary taps” in Figure 4, allow the chamber to be emptied.

53. Transfer the setup to an incubator set to 42°C.
54. Following the black and blue arrows in Figure 4, secure the tubing from the chamber to the Ismatec pump via a pump cassette, and vice versa.

***Note:*** The three-way Luer locks, referred to as the “primary taps” in Figure 4, allow the chamber to be filled.

55. Place a 0.2 μm membrane filter in the position marked “air filter”, attaching via a short silicone tube.

***Note:*** The flow rig now has all the tubes attached and should now match the schematic presented in Figure 4.

56. Add PBS with 1× P/S to the chamber via the primary tap with a 50 mL syringe, slowly depressing the syringe with a small amount of pressure.

***Note:*** The solution should perfuse through the right ventricle, past the pulmonary valve, and into the artery.

**CRITICAL:** It is important to observe flow following the direction of the arrows indicated in Figure 4. Solution should be perfused from the primary tap, through the lumen of the RVOT, past the secondary tap, and then into the sample chamber, demonstrating a closed system wherein the sample is perfused past the pulmonary valve. Once the sample chamber is full, the solution will flow into the reservoir chamber.

57. Continue filling with a syringe until the reservoir chamber is approximately half full.

***Note:*** The volume of solution is determined by the RVOT and chamber size. Sufficient volume should be used to completely fill the sample chamber and a minimum of half the reservoir chamber.

58. Set the pump to a constant rate of 32 mL/min (50 rpm on the pump model indicated in the key resources table).

**CRITICAL:** Ensure the direction of flow is from the ventricle to the artery, through the pulmonary valve.

59. Leave the PBS with 1× P/S wash for 1 h to ensure appropriate flow and no leaks. Troubleshooting, problem 2.

#### Part 3d: Decellularization procedure

**Timing: total timing: 9 days, hands-on-time: 5 h, variable**

**Figure 5:**
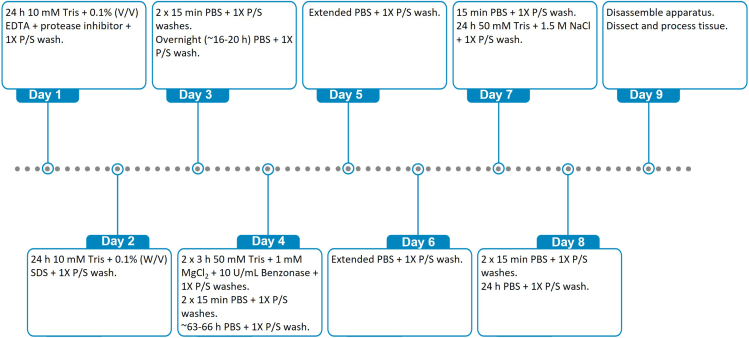
Timeline of the decellularization protocol with solution changes indicated

To decellularize the RVOTs, samples are exposed to an eight-day decellularization procedure (Figure 5). This method achieves a decellularized scaffold, validated in part 4: characterization of decellularized tissue.

**CRITICAL:** Solutions are filtered with a 0.2 μm membrane filter prior to use if the working solution includes any component not already sterilized.

***Note:*** For each solution change, volumes are given to fill a 250 mL flow chamber, but this can be scaled according to the chamber volume.

60. Decellularization day 1 (1 solution change).
  a. In a sterile 250 mL beaker, make 0.1% (v/v) ethylenediaminetetraacetic acid (EDTA) with Pierce A32965 protease inhibitor in 10 mM Tris buffer and 1× P/S.
    i. Add 250 μL 0.5 M EDTA to a sterile 250 mL beaker.
    ii. Add 5 tablets Pierce A32965 protease inhibitor.
    iii. Add 2.5 mL 100× P/S stock.
    iv. Make up to 250 mL with 10 mM Tris (RVOT decellularization solution 1).
    v. Filter sterilize with a 0.2 μm membrane filter.
      ***Note:*** EDTA is a chelating agent that disrupts cell adhesions.
  b. Remove the solution from the chamber.
    ***Note:*** To remove solution from the chamber, the reservoir chamber is emptied via both primary taps with a 50 mL syringe. The sample chamber is emptied via both secondary taps.
  c. Fill the chamber with the solution prepared in step 60a.
  d. Leave the solution circulating for 24 h at 42°C.
61. Decellularization day 2 (1 solution change).
  a. In a sterile 250 mL beaker, make 0.1% (w/v) SDS in 10 mM Tris buffer and 1× P/S.
    i. Add 2.5 mL 10% (w/v) SDS stock (RVOT decellularization solution 2) to a sterile 250 mL beaker.
    ii. Add 2.5 mL 100× P/S stock.
    iii. Make up to 250 mL with 10 mM Tris.
    iv. Filter sterilize with a 0.2 μm membrane filter.
  b. Remove the solution from the chamber.
  c. Fill the chamber with the solution prepared in step 61a.
  d. Leave the solution circulating for 24 h at 42°C.
62. Decellularization day 3 (3 solution changes).
  a. In a sterile 1 L beaker, make 1× PBS with 1× P/S
    i. Add 7.5 mL 100× P/S stock to a sterile 1 L beaker.
    ii. Make up to 750 mL with 1× PBS.
  b. Remove the solution from the chamber.
  c. Fill the chamber with 250 mL of the solution prepared in step 62a.
  d. Leave the solution circulating for 15 min at 42°C.
  e. Remove the solution from the chamber.
  f. Fill the chamber with 250 mL of the solution prepared in step 62a.
  g. Leave the solution circulating for 15 min at 42°C.
  h. Remove the solution from the chamber.
  i. Fill the chamber with 250 mL of the solution prepared in step 62a.
  j. Leave the solution circulating overnight (16–20 h) at 42°C.
63. Decellularization day 4 (5 solution changes).
  a. In a sterile 250 mL beaker, make 10 U/mL Benzonase in 50 mM Tris, 1 mM MgCl_2_, and 1× P/S.
    ***Note:*** The enzyme activity of Benzonase will be slightly variable between batches. Check the certificate of analysis for each batch and adjust the volume of Benzonase added to produce a solution of 10 U/mL. Below is an example calculation for a Benzonase stock solution of 26.6 U/μL.
    i. Add 94 μL 26.6 U/μL Benzonase stock to a sterile 250 mL beaker.
    ii. Add 2.5 mL 100× P/S stock.
    iii. Make up to 250 mL with 50 mM Tris + 1 mM MgCl_2_ (RVOT decellularization solution 3).
    iv. Filter sterilize with a 0.2 μm membrane filter.
  b. Remove the solution from the chamber.
  c. Fill the chamber with the solution prepared in step 63a.
  d. Leave the solution circulating for 3 h at 37°C.
  e. Repeat step 63a-d.
  f. In a sterile 1 L beaker, make 1× PBS with 1× P/S.
    i. Add 7.5 mL 100× P/S stock to a sterile 1 L beaker.
    ii. Make up to 750 mL with 1× PBS.
  g. Remove the solution from the chamber.
  h. Fill the chamber with 250 mL of the solution prepared in step 63f.
  i. Leave the solution circulating for 15 min at 42°C.
  j. Remove the solution from the chamber.
  k. Fill the chamber with 250 mL of the solution prepared in step 63f.
  l. Leave the solution circulating for 15 min at 42°C.
  m. Remove the solution from the chamber.
  n. Fill the chamber with 250 mL of the solution prepared in step 63f.
  o. Leave the solution circulating for ∼63–66 h at 42°C.
    ***Note:*** Days 5 and 6 of the decellularization are part of the extended 2-day PBS with 1× P/S wash (42°C), involving no solution changes.
64. Decellularization day 7 (2 solution changes).
  a. In a sterile 250 mL beaker, make 1× PBS with 1× P/S.
    i. Add 2.5 mL 100× P/S stock to a sterile 250 mL beaker.
    ii. Make up to 250 mL with 1× PBS.
  b. Remove the solution from the chamber.
  c. Fill the chamber with the solution prepared in step 64a.
  d. Leave the solution circulating for 15 min at 42°C.
  e. In a sterile 250 mL beaker, make 50 mM Tris, 1.5 M NaCl, and 1× P/S.
    i. Add 2.5 mL 100× P/S stock to a sterile 250 mL beaker.
    ii. Make up to 250 mL with 50 mM Tris and 1.5 M NaCl (RVOT decellularization solution 4).
  f. Remove the solution from the chamber.
  g. Fill the chamber with the solution prepared in step 64e.
  h. Leave the solution circulating for 24 h at 42°C.
65. Decellularization day 8 (3 solution changes).
  a. In a sterile 1 L beaker, make 1× PBS with 1× P/S.
    i. Add 7.5 mL 100× P/S stock to a sterile 1 L beaker.
    ii. Make up to 750 mL with 1× PBS.
  b. Remove the solution from the chamber.
  c. Fill the chamber with 250 mL of the solution prepared in step 65a.
  d. Leave the solution circulating for 15 min at 42°C.
  e. Remove the solution from the chamber.
  f. Fill the chamber with 250 mL of the solution prepared in step 65a.
  g. Leave the solution circulating for 15 min at 42°C.
  h. Remove the solution from the chamber.
  i. Fill the chamber with 250 mL of the solution prepared in step 65a.
  j. Leave the solution circulating for overnight (16–20 h) at 42°C.
66. Disassemble the apparatus and clean the chamber.
  a. Remove the final PBS with 1× P/S wash solution and discard.
  b. Disconnect the chamber from the tubing.
  c. Spray the external surfaces of the chamber with 70% (v/v) ethanol and move the chamber to the class II microbiological safety cabinet.
  d. Remove clamps and take off the lid and gasket.
  e. Remove the decellularized (d)RVOT from the chamber by disconnecting the inner tubes.
  f. Carefully cut the sutures to release the dRVOT from the hose barbs.
  g. Place the dRVOT in autoclaved PBS with 1× P/S.
  h. Disassemble all tubes, connectors, and hose barbs, placing small parts into a suitably sized box to prevent loss.
  i. Discard the taps.

***Note:*** The dRVOT is now ready for characterization.

**CRITICAL:** Carry out full apparatus sterilization as described in part 3a: sterilize the apparatus. Troubleshooting, problem 3. Troubleshooting, problem 4.

### Part 4: Characterization of decellularized tissue

**Timing: total timing: 1 day, hands-on-time: 1 h (for step 67)**

**Timing: total timing: 3 days, hands-on-time: 2 h per (d)RVOT, variable (for step 68)**

**Timing: 1 day (for step 69)**

**Timing: 1 day (for step 70)**

**Timing: total timing: 2 days, hands-on-time: 3 h per RVOT (for step 71)**

**Timing: 2 h per RVOT (for step 72)**

**Timing: 1 h (for step 73)**

**Timing: 1 h per RVOT (for step 74)**

This section refers to the initial characterization steps essential to validate the decellularization process. Following decellularization, the tissue is immediately processed for characterization by histology, IHC, SEM, DNA isolation/quantification, and tensile testing to evaluate cell removal and maintenance of the ECM. To interrogate the impact of freezing on protocol success, characterization of the right ventricle, pulmonary artery, and pulmonary valve leaflet was carried out on four groups^1^: Fresh RVOTs,^2^ freeze-thawed (F-T) RVOTs,^3^ fresh decellularized (d)RVOTs, and^4^ freeze-thawed decellularized (F-T d)RVOTs.

**Figure 6:**
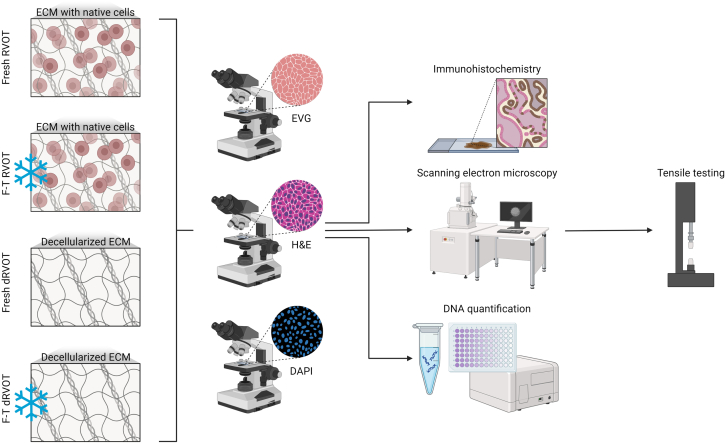
Characterization steps to validate the decellularization process Histological assessment (hematoxylin and eosin (H&E), elastin Van Gieson (EVG), and 4′,6-diamidino-2-phenylindole (DAPI)) was carried out on all fresh native samples (fresh RVOT), freeze-thawed native samples (F-T RVOT), fresh decellularized samples (fresh dRVOT), and freeze-thawed decellularized samples (F-T dRVOT) as quality control. Following this, a subset was taken forward for further evaluation by immunohistochemistry, scanning electron microscopy, and DNA quantification. Tensile testing was carried out on F-T RVOTs and F-T dRVOTs.

***Note:*** Once the success of the protocol has been validated by the individual replicating this method, much of part 4 can be omitted and dRVOTs can be kept whole for downstream application (e.g., cell seeding). However, quality control of each dRVOT should always be carried out by processing a small section (5 mm^2^) of the pulmonary artery and right ventricle from each dRVOT for basic histological assessment (hematoxylin and eosin (H&E), elastin Van Gieson (EVG), and 4′,6-diamidino-2-phenylindole (DAPI) staining). However, we also recommend carrying out full quality control assessment at regular intervals by conducting the complete characterization (Figure 6) as described in part 4: characterization of decellularized tissue.

***Note:*** For readability, these characterization steps are described based on processing a single dRVOT. However, the same approach is applied to control tissue for comparison and to validate success.

67. Dissection and fixation/storage immediately following decellularization (Figure 7).
  ***Note:*** Once the apparatus has been disassembled, the dRVOT is dissected for analysis. Given tissue limitations, we recommend prioritizing the below characterization, however this is not an exhaustive list of possibilities to determine whether the scaffold is successfully decellularized.
  a. dRVOT processing to formaldehyde-fixed tissue for histology and IHC.
    i. Fill the wells of a 96-well plate with 4% (w/v) formaldehyde.
      ***Note:*** PFA is a hazardous reagent. Follow the manufacturer’s guidance on how to handle and store PFA. Wear personal protection equipment (lab coat, gloves, and eye protection).
    ii. Using sterile forceps, transfer 5 mm^2^ central biopsies of right ventricle, 5 mm^2^ central biopsies of pulmonary artery, and approximately half of a pulmonary valve leaflet to the 96-well plate.
      **CRITICAL:** Ensure the tissue is fully submerged in the formaldehyde solution.
    iii. Fix the tissue overnight (16–20 h) at 4°C.
  b. dRVOT processing to glutaraldehyde/sodium cacodylate-fixed tissue for SEM.
    i. Mix 2 mL of the 0.2 M sodium cacodylate stock and 2 mL 5% (v/v) glutaraldehyde stock in a 15 mL falcon tube.
      ***Note:*** Glutaraldehyde and sodium cacodylate are hazardous reagents. Follow the manufacturer’s guidance on how to handle and store these. Wear personal protection equipment (lab coat, gloves, and eye protection). Always prepare working solution freshly.
    ii. Fill the wells of a 96-well plate with the 2.5% (v/v) glutaraldehyde in 0.1 M sodium cacodylate working solution.
    iii. Using sterile forceps, transfer 5 mm^2^ central biopsies of right ventricle, 5 mm^2^ central biopsies of pulmonary artery, and approximately half a pulmonary valve leaflet to the 96-well plate.
      **CRITICAL:** Ensure the tissue is fully submerged in the glutaraldehyde/sodium cacodylate solution.
    iv. Fix the tissue overnight (16–20 h) at 4°C.
      ***Note:*** A longer fixation period with 2.5% (v/v) glutaraldehyde in 0.1 M sodium cacodylate will not negatively affect the tissue, as long as the samples are kept sterile. Up to 7 weeks of glutaraldehyde in sodium cacodylate fixation was tested, though fixation for a longer period may be possible.
  c. Aliquot tissue for DNA isolation and quantification.
    i. Using sterile forceps, transfer right ventricle (∼50 mg/vial; two vials), pulmonary artery (∼50 mg/vial; two vials), and pulmonary valve (1 leaflet/vial; two vials) into cryovials.
    ii. Freeze samples at −80°C.
    iii. Place the remaining pulmonary artery in PBS for tensile testing. Store at 4°C and use the same day.
    iv. Discard the remaining right ventricle.

**Figure 7:**
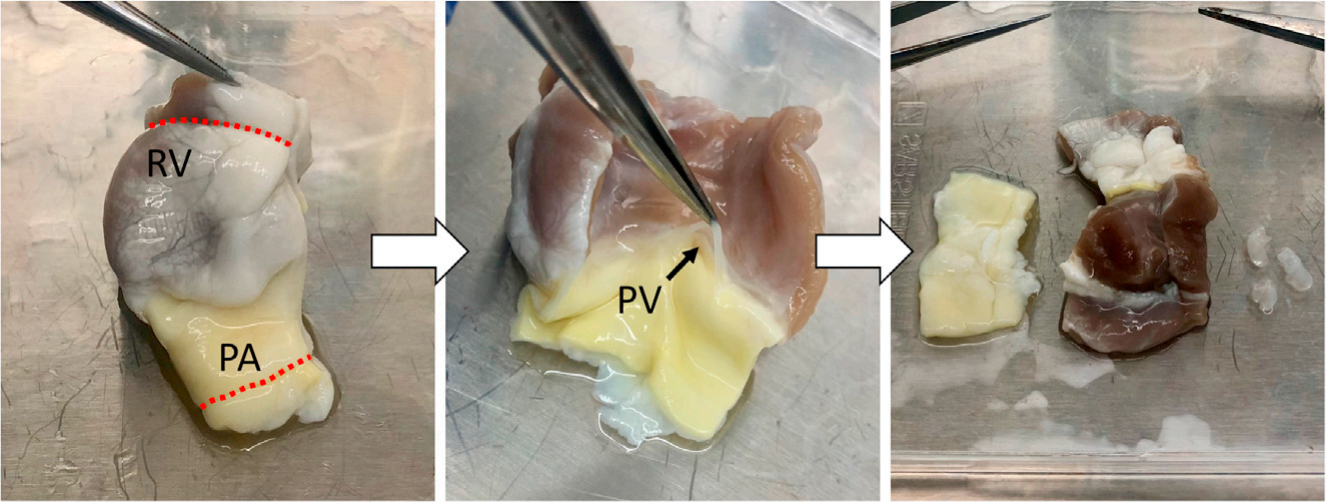
Dissection of the decellularized RVOT First, remove the area that was under the suture for the duration of decellularization, marked in red. Next, remove the pulmonary valve (PV) leaflets, then separate the pulmonary artery (PA) from the right ventricle (RV).
68. Basic histological characterization of formaldehyde-fixed paraffin-embedded tissue.
  a. Following fixation in 4% (w/v) formaldehyde, place samples in histology cassettes and submerge in PBS. Store at 4°C.
  b. Perform an automated dehydration series.
    ***Note:*** Here we used a Thermo Excelsior AS on a 9 h program.
  c. Embed samples longitudinally in paraffin wax.
    ***Note:*** Here we used a Thermo HistoStar machine.
    **CRITICAL:** The orientation of the sample is vital for histological analysis. Cross sections are required to interrogate the structures through the depth of the tissue.
    **Pause point:** At this point, paraffin-embedded tissue can be stored at room temperature (usually 18°C–22°C) for many years.
  d. Once cooled, remove the block of paraffin embedded tissue.
  e. Cut serial 5 μm thick sections longitudinally on a microtome.
  f. Mount sections on Menzel-Glaser SuperFrost Plus slides.
    ***Note:*** We recommend mounting a minimum of 3 sections per slide, where space allows, and preparing a minimum of 5 slides per sample.
  g. Store mounted sections at 37°C overnight (16–20 h) to dry.
  h. Perform standard histological staining procedures for H&E, EVG, and DAPI.
  i. Leave samples to dry overnight (16–20 h) before imaging.
    ***Note:*** Here, sections were stained with H&E and EVG using a Shandon Varistain 24-4 autostainer. Slides were mounted with a coverslip and DPX Mountant. For DAPI, sections were manually deparaffinized by placing in three 5 min changes of Clearene. Sections were then rehydrated in four changes of gradated ethanol series (100% (v/v), 100% (v/v), 90% (v/v), 70% (v/v)) for 5 min each. Sections were dried and mounted with a drop of ProLong Gold Antifade Mountant with DAPI. Images were captured with 10×, 20×, and 40× objectives in three distinct regions per sample using a Zeiss Axio Observer.Z1 and Zen Blue software or the Olympus VS200 slide scanner and OlyVIA software.
    ***Note:*** though nuclei were also quantified from H&E images, we do not recommend this approach. The degree of error is far greater in comparison to DAPI quantification, established through comparison of automated DAPI or H&E image processing with three independent manual counts of both control and decellularized tissue.
69. IHC of formaldehyde-fixed paraffin-embedded tissue for the a-Gal epitope.
  ***Note:*** IHC is carried out at room temperature (usually 18°C–22°C) unless otherwise stated.
  a. Heat slides at 60°C for 20 min to ensure tissue sections are well adhered to the slide.
  b. Place slides in a staining rack.
  c. Deparaffinize sections by placing in three 5 min changes of Clearene.
  d. Rehydrate sections in four changes of gradated ethanol series (100% (v/v), 100% (v/v), 90% (v/v), 70% (v/v)) for 5 min each.
  e. Rinse slides in dH_2_O.
  f. Carry out a microwave antigen retrieval method:
    i. Heat a full trough of 10 mM citrate buffer (pH 6.0) at full power for 4 min.
    ii. Place full staining rack in the hot citrate buffer and top up to full.
    iii. Microwave for 4 min at full power (800 W).
    iv. Top up citrate buffer to full if necessary.
    v. Heat for 4 min at full power.
    vi. Leave to cool for 30 min.
  g. On a rocker, wash slides three times in 1× PBS for 5 min each.
  h. Carefully dry slides, avoiding contact with the tissue, and circle sections with an ImmEdge Pen.
  i. Inhibit endogenous peroxidase with Bloxall for 15 min in a humidified chamber.
  j. On a rocker, wash slides three times in 1× PBS for 5 min each.
  k. Block nonspecific antibodies for 1 h with 10% (v/v) normal horse serum in a humidified chamber.
  l. Carefully dry slides and incubate sections with 1:5 α-gal epitope monoclonal primary antibody M86 in 10% (v/v) normal horse serum for 1 h in a humidified chamber. Incubation with 1× PBS serves as a negative control.
  m. On a rocker, wash slides three times in 1× PBS for 5 min each.
  n. Carefully dry slides and incubate sections with 1:250 biotin-labeled goat polyclonal anti-mouse IgM secondary antibody in 10% (v/v) normal horse serum for 30 min in a humidified chamber.
  o. On a rocker, wash slides three times in 1× PBS for 5 min each.
  p. Carefully dry slides and incubate sections with 1:200 extravidin-horseradish peroxidase in 10% (v/v) normal horse serum for 30 min in a humidified chamber.
  q. On a rocker, wash slides three times in 1× PBS for 5 min each.
  r. Carefully dry slides and incubate sections with 3,3′-diaminobenzidine (DAB), prepared according to the manufacturer’s instructions (DAB substrate kit protocol), for up to 10 min for color development.
    i. For right ventricle and pulmonary valve leaflets, sections were incubated for 4 min.
    ii. For pulmonary artery, sections were incubated for 6 min.
  s. Wash sections well in tap water.
  t. Counterstain sections for 1 min with filtered 50:50 MAYER hematoxylin/dH_2_O.
  u. Wash sections under running tap water for 5 min.
    i. Let trough fill and empty once.
    ii. Leave to overflow for the remaining time.
  v. Dehydrate sections in four changes of gradated ethanol series (70% (v/v), 90% (v/v), 100% (v/v), 100% (v/v)) for 5 min each.
  w. Remove ethanol by placing in three 5 min changes of Clearene.
  x. Mount coverslip with DPX mountant.
  y. Leave samples to dry overnight (16–20 h) before imaging.
    ***Note:*** Images were captured with the Olympus VS200 slide scanner and OlyVIA software.
70. SEM.
  ***Note:*** SEM is carried out at room temperature (usually 18°C–22°C) unless otherwise stated.
  a. Following fixation, wash samples once in 0.1 M sodium cacodylate for 5 min.
  b. Wash samples twice in dH_2_O for 5 min.
  c. Dehydrate samples using serial ethanol washes for 5 min each (30% (v/v), 50% (v/v), 70% (v/v), 90% (v/v), 96% (v/v)).
  d. Wash samples twice in 100% (v/v) ethanol for 10 min.
  e. Place samples in a holder, separated by glass coverslips, keeping them submerged in ethanol.
  f. Dry samples in a critical point dryer on a slow 15 cycle program.
  g. Bisect the dried pulmonary artery section in half, turning one over to expose the opposite surface.
    ***Note:*** It is possible to distinguish the inner pulmonary artery luminal surface from the outer adventitial surface, with the former being uniform and smooth, and the latter slightly rougher.
  h. Mount the samples on stubs with carbon tabs.
  i. Sputter coat samples with Au/Pd for 1 min at 40 mA.
    **Pause point:** Samples can be stored indefinitely following sputter coating if they are protected from debris.
    ***Note:*** Surface topography was imaged on a Quanta 200 FEI field emission scanning electron microscope. A minimum of three locations at five magnifications were viewed using the Everhart-Thornley detector, a secondary electron and back scattered electron detector.
71. DNA isolation.
  ***Note:*** With the exception of the pulmonary valve leaflet, which could not be removed prior to decellularization, DNA was extracted from biologically matched samples. Where tissue availability allowed, DNA was isolated from technical duplicates for each animal and tissue.
  ***Note:*** A DNeasy Blood and Tissue Kit was used for DNA extraction. A maximum of 25 mg of tissue was used per extraction, in line with the manufacturer’s guidelines (DNeasy Blood & Tissue Handbook).
  **CRITICAL:** DNA should always be isolated from control samples in parallel with decellularized samples.
  a. Cool the freeze dryer platform to <−60°C.
  b. Defrost biologically matched right ventricle and decellularized right ventricle, matched pulmonary artery and decellularized pulmonary artery, and unmatched pulmonary valve leaflet and decellularized pulmonary valve leaflet aliquots on ice for 30 min.
  c. Using sterile forceps and surgical scissors, dissect tissue into small pieces, keeping samples on ice as much as possible.
  d. For each sample, weigh approximately 20 mg wet tissue onto a weighing boat.
    **CRITICAL:** Use scales that read to a mass accuracy of 0.1 mg.
    ***Note:*** Where possible, duplicates should be weighed (i.e., for the samples in step 71d, it is expected that there will be 2 x right ventricle, 2 x decellularized right ventricle, 2 x pulmonary artery, 2 x decellularized pulmonary artery, 1 x pulmonary valve leaflet, and 1 x decellularized pulmonary valve leaflet, with each tissue type in a separate weighing boat).
  e. Transfer the weigh boats to the freeze drying platform and freeze dry for 2 h.
  f. Weigh the dry mass of each sample three times and record the average of the three dry weights.
    **CRITICAL:** Use scales that read to a mass accuracy of 0.1 mg.
    ***Note:*** The tissue is now ready for DNA isolation using the Spin-Column protocol from the DNeasy Blood & Tissue Handbook.
    ***Note:*** All steps of the purification of total DNA from porcine tissue are carried out at room temperature (usually 18°C–22°C) unless otherwise stated and the flow-through discarded, except for the elution with AE buffer.
  g. Preheat a heat block to 56°C.
  h. Place freeze dried tissue in a DNase/RNase free 1.5 mL reaction tubes.
  i. Add 180 μL ATL buffer and 20 μL Proteinase K.
  j. Pulse-vortex samples for 10 s and incubate at 56°C for 20–22 h to ensure full lysis.
  k. Vortex samples for a minimum of 10 s.
  l. Heat sufficient AE buffer to 60°C in a heat block.
    ***Note:*** 1 mL AE buffer is needed for each sample.
  m. Add 200 μL AL buffer and 200 μL 96–100% (v/v) ethanol.
    ***Note:*** A white precipitate may form, however this does not affect DNA isolation.
  n. Thoroughly vortex samples to yield a homogenous solution, dispersing any precipitate.
  o. Transfer the solution to a DNeasy Mini spin column in a 2 mL collection tube.
  p. Briefly centrifuge the reaction tubes to recover any remaining solution and add the remaining solution to the relevant collection tube.
  q. Centrifuge the spin columns at 6000 *g* for 1 min at room temperature (usually 18°C–22°C).
  r. Discard the collection tube with the flow through, transferring the spin column to a new collection tube.
  s. Add 500 μL AW1 buffer to the spin columns.
    **CRITICAL:** Do not use AW1 buffer in its concentrated form. The AW1 buffer concentrate must first be diluted with 25 mL 96–100% (v/v) ethanol.
  t. Centrifuge the spin columns at 6000 *g* for 1 min at room temperature (usually 18°C–22°C).
  u. Discard the collection tube with the flow through, transferring the spin column to a new collection tube.
  v. Add 500 μL AW2 buffer to the spin columns.
    **CRITICAL:** Do not use AW2 buffer in its concentrated form. The AW2 buffer concentrate must first be diluted with 30 mL 96–100% (v/v) ethanol.
  w. Centrifuge the spin columns at 20000 *g* for 3 min at room temperature (usually 18°C–22°C) to dry the membrane.
  x. Place the column in a DNase/RNase-free 1.5 mL reaction tube and add 200 μL 60°C AE buffer onto the membrane.
  y. Incubate the sample for 5 min and then centrifuged at 6000 *g* for 1 min at room temperature (usually 18°C–22°C).
  z. Repeat steps 71x-y a further four times, each into a new reaction tube, resulting in five 200 μL eluates.
    ***Note:*** DNA levels of decellularized eluants are below the detection limit of standard nanospectrophotometers due to the extremely low concentration of nucleic acids in the decellularized samples. Standard nanospectrophotometers, for example the ND-1000, have a detection limit of 2 ng/μL, whereas the PicoGreen assay can detect as little as 0.25 pg/μL of double stranded (ds)DNA.
    ***Note:*** In accordance with the paper often used as a reference point for the minimum criteria for decellularization,^2^ dsDNA should be quantified. Therefore, the following quantification method is suggested, beginning with elimination of single stranded nucleic acid contaminants by P1 enzyme digestion.
72. Elimination of single stranded nucleic acid contaminants.
  ***Note:*** Once the DNA has been eluted into five 200 μL volumes of AE buffer, elimination of RNA and/or single stranded DNA by nuclease P1, a single stranded-specific endonuclease, enables accurate quantification of total dsDNA by PicoGreen.
  ***Note:*** Though the PicoGreen dsDNA assay is sensitive for quantifying dsDNA in the presence of single stranded nucleic acids, single stranded nucleic acids can interfere with the signal intensity. This is particularly important to account for when measuring very low concentrations of dsDNA, as is the case for decellularized eluants. For example, 50 ng/mL single stranded DNA in 500 ng/mL dsDNA (0.1× single stranded DNA relative to dsDNA) can result in up to a 10% increase in the sample’s signal intensity (PicoGreen assay, page 5).
  a. Preheat two heat blocks, one to 37°C and one to 75°C.
  b. Prepare 20 mL 1× TE buffer by diluting 1 mL 20× stock (provided in the PicoGreen kit) in 19 mL DNase/RNase free water.
  c. Combine elutions (e)1-5 in a DNase/RNase free microtube.
    i. For control samples, combine 3 μL of each elution.
    ii. For decellularized samples, combine 28 μL of each elution.
  d. Vortex to thoroughly mix.
  e. Dilute e1-5 in 1× TE buffer.
    i. For control samples, add 10 μL e1-5 to 240 μL 1× TE (4% (v/v) DNA).
    ii. For decellularized samples, add 125 μL e1-5 to 125 μL 1× TE (50% (v/v) DNA).
  f. Vortex to thoroughly mix.
  g. Add 44.9 μL diluted e1-5 to five DNase/RNase free microtubes per sample.
  h. Add 0.1 μL nuclease P1 and 5 μL NE buffer r1.1 to each tube.
    ***Note:*** We suggest making a master mix to avoid pipetting volumes <1 μL.
    **CRITICAL:** When calculating the true concentration of each sample in step 73. Quantification of double stranded DNA, ensure the addition of nuclease P1 and NE buffer r1.1 is accounted for (Table 1).

**Table 1:** Example of raw and processed data measured on a plate reader

Sample	Raw data		Processed data				
	Replicate 1 absorbance	Replicate 2 absorbance	Avg. absorbance	Corrected avg. absorbance	Concentration/ ng/mL	True concentration/ ng/mL	Mass of DNA per mg dry tissue/ ng/mg
			Average of replicate 1 and 2 absorbance	Subtract average of the 8 blanks	Standards: known concentration. Unknowns: use the equation of the line	Account for dilution factor	Divide by known dry tissue mass
Blank	20.04859086	19.82692863	20.16199324	N/A			
Blank	19.75195001	19.78419527		N/A			
Blank	20.14641058	21.06173078		N/A			
Blank	20.16349199	20.51264778		N/A			
Standard 1 (0 ng/mL)	Use the average of the 8 blanks		20.16199324	0.00000000	0	0	N/A
Standard 2 (0.5 ng/mL)	22.56963662	22.3992901	22.48446336	2.32247012	0.5	0.5	
Standard 3 (2.5 ng/mL)	34.3947504	33.84805076	34.12140058	13.95940734	2.5	2.5	
Standard 4 (10 ng/mL)	76.23690786	75.38246334	75.80968560	55.64769236	10	10	
Standard 5 (25 ng/mL)	158.5718667	157.7199527	158.14590968	137.98391644	25	25	
Standard 6 (50 ng/mL)	310.4586	307.0832855	308.77094276	288.60894952	50	50	
Standard 7 (150 ng/mL)	839.2340431	882.0724001	860.65322156	840.49122832	150	150	
Standard 8 (300 ng/mL)	1709.299305	1723.141434	1716.22036929	1696.05837605	300	300	
Standard 9 (500 ng/mL)	2858.500352	2854.54476	2856.52255583	2836.36056260	500	500	
Equation of the line	Y = 5.6678x – 1.3075; R^2^ = 1						
Unknown 1 (e.g., right ventricle)	1035.073692	1061.042273	1048.05798213 (Average)	1027.89598890 (Average – blanks)	181.58710077 ((Corrected average absorbance – 1.3075)/5.6678)	5055.32017739 (Divide by 0.03592, the dilution factor of the nuclease P1 digested sample)	1444.37719354 (Divide by 3.5 mg, the starting dry tissue mass)
Unknown 2 (e.g., decellularized right ventricle)	212.3037226	210.8356375	211.56968005 (Average)	191.40768681 (Average – blanks)	34.00161910 ((Corrected average absorbance – 1.3075)/5.6678)	75.72743675 (Divide by 0.449, the dilution factor of the nuclease P1 digested sample)	31.55309865 (Divide by 2.4 mg, the starting dry tissue mass)
  i. Vortex to thoroughly mix.
  j. Incubate the samples at 37°C in a heat block for 30 min.
  k. Deactivate the P1 enzyme at 75°C in a heat block for 10 min.
  l. Leave the samples to cool.
  m. Combine the five aliquots into one microtube per sample.
73. Quantification of double stranded DNA.
  ***Note:*** Following elimination of single stranded nucleic acid contaminants, dsDNA can be quantified by a PicoGreen assay, according to the manufacturer’s instructions (PicoGreen assay).
  a. Dilute 6 μL 100 μg/mL dsDNA standard 50-fold in 294 μL 1× TE to prepare the 2 μg/mL dsDNA stock solution.
  b. Dilute 10 μL 2 μg/mL dsDNA standard 40-fold in 390 μL 1× TE to prepare the 50 ng/mL dsDNA stock solution.
  c. Prepare 250 μL of each dsDNA standard as follows:

**Table:** 

Volume 1× TE/ μL	Stock DNA concentration/ng/mL	Volume stock/μL	Concentration/ng/mL	Final DNA concentration in assay/ng/mL
900	N/A	0	0	0
245	50	5	1	0.5
225	50	25	5	2.5
150	50	100	20	10
0	50	250	50	25
237.5	2000	12.5	100	50
212.5	2000	37.5	300	150
175	2000	75	600	300
125	2000	125	1000	500
  d. Prepare the PicoGreen dsDNA working reagent by diluting the stock dsDNA Quant-iT PicoGreen reagent (warmed to room temperature, usually 18°C–22°C) 200-fold in 1× TE.
    ***Note:*** For microplate assays of a total 200 μL assay volume, 100 μL of PicoGreen dsDNA working reagent is needed per measurement. E.g., for 50 samples, 5 mL can be prepared by combining 25 μL of reagent with 4.975 mL of 1× TE buffer.
    **CRITICAL:** Protect the PicoGreen dsDNA working reagent from light and use the solution within a few hours of preparation.
  e. Vortex all standards and samples to mix thoroughly.
  f. Add 100 μL of each dsDNA standard and unknown experimental dsDNA sample to a black 96-well plate, plating in duplicate.
    **CRITICAL:** Given the extremely low levels of dsDNA in the decellularized samples, we recommend carrying out the PicoGreen assay with 8 blanks, with duplicates in each corner of the plate. These can then be averaged and used to normalize the readings and minimize the effect of variability across the plate (Table 1).
  g. Add 100 μL of the PicoGreen dsDNA working reagent to each well, making a total of 200 μL/well.
  h. Mix well and incubate at room temperature (usually 18°C–22°C) for 2–5 min while protecting from light.
  i. Read the fluorescence of each well by setting the plate reader to an excitation wavelength of 480 nm and emission wavelength of 520 nm.
    ***Note:***Table 1 shows a worked example of the data analysis methodology to process the raw data measured on a plate reader.
    ***Note:*** The assay can be repeated using a different dilution of the unknown sample to confirm the quantification results. Troubleshooting, problem 5.
74. Evaluation of tensile properties.
  ***Note:*** Mechanical characterization of the scaffolds was performed using an Instron 3343 and a 100 N load cell. Other tensile testing equipment is also suitable, such as the Instron 6800 and 3400 Series electromechanical universal testing machines.
  a. Turn on the compressor and Instron machine, ensuring the load cell arms are aligned.
  b. Setup the Bluehill 3 software with the following input parameters:
    i. Young’s Modulus.
    ii. Load at break.
    iii. Stress at maximum load.
    iv. Summary of dimensions.
  c. Cut the pulmonary artery into four uniform 15 × 5 mm rectangular pieces with a razor blade, longitudinally from the ventricle.
    **CRITICAL:** Keep samples in PBS until taking measurements.
  d. Measure the width and thickness of each sample at the halfway point with a micrometer and vernier calipers, respectively.
  e. Clamp the sample in the pneumatic grips in the longitudinal direction with an initial displacement of 5 mm.
  f. Move the arm up until the sample is straight.
  g. Measure the length of the sample with vernier calipers.
  h. Input the dimensions.
  i. Balance the load and zero the extension.
  j. Set the steady deformation rate of 10 mm/min.
  k. Begin the tensile test.
  l. Stop the test when the curve falls below 50% of the maximum peak.
  m. Remove the sample from the pneumatic grips.
  n. Repeat steps 74e-m for the following replicates.
    **CRITICAL:** If the sample breaks at the level of the clamps, the data is not trustworthy and should be excluded from analysis.

## Expected outcomes

**Figure 8:**
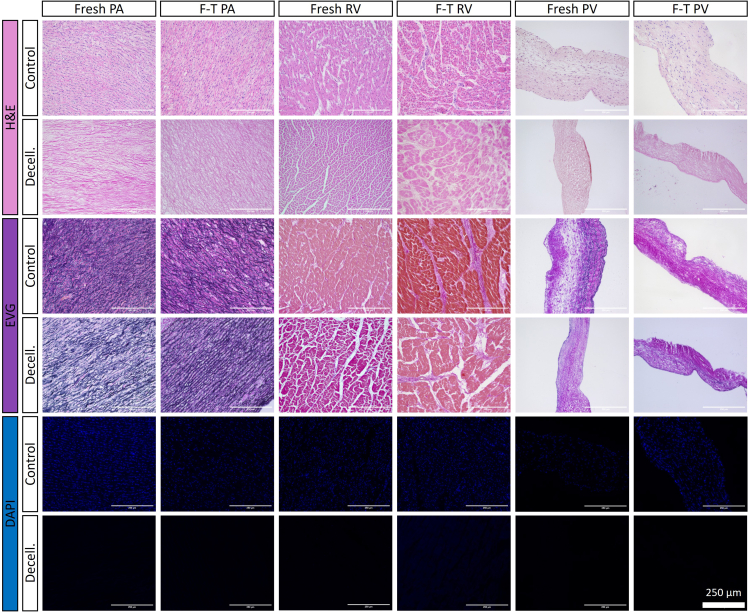
Representative hematoxylin and eosin (H&E), elastin Van Gieson (EVG), and 4′,6-diamidino-2-phenylindole (DAPI) staining of fresh and freeze-thawed (F-T) control and decellularized (decell.) right ventricle (RV), pulmonary artery (PA), and pulmonary valve (PV) leaflet cross sections Following decellularization, nuclei are not observed in the H&E or DAPI staining. The elastin and collagen fibers, depicted in the EVG, appear intact. Fresh RVOT, n = 5; F-T RVOT, n = 5; fresh dRVOT, n = 6; F-T dRVOT, n = 9. Scale bar = 250 μm.

**Figure 9:**
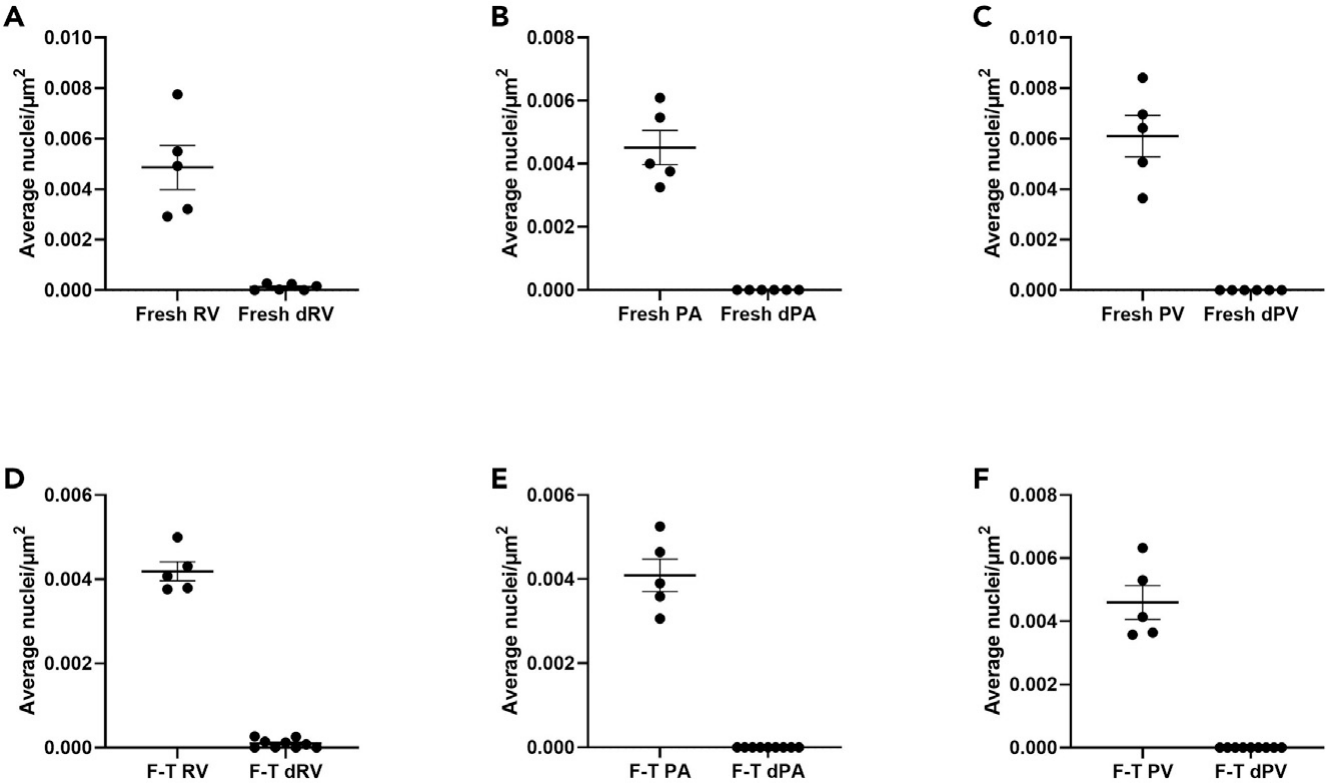
Quantification of DAPI staining Each point represents the mean nuclei count across five regions of interest from the DAPI stained section. (A) Fresh right ventricle (RV) versus fresh decellularized right ventricle (dRV). (B) Fresh pulmonary artery (PA) versus fresh decellularized pulmonary artery (dPA). (C) Fresh pulmonary valve (PV) leaflet versus fresh decellularized pulmonary valve (dPV) leaflet. (D) Freeze-thawed right ventricle (F-T RV) versus freeze-thawed decellularized right ventricle (F-T dRV). (E) Freeze-thawed pulmonary artery (F-T PA) versus freeze-thawed decellularized pulmonary artery (F-T dPA). (F) Freeze-thawed pulmonary valve (F-T PV) leaflet versus freeze-thawed decellularized pulmonary valve (F-T dPV) leaflet. Graphs show the mean ± SEM from the depicted tissue type. Fresh RVOT, n = 5; fresh dRVOT, n = 6; F-T RVOT, n = 5; F-T dRVOT, n = 9.

**Figure 10:**
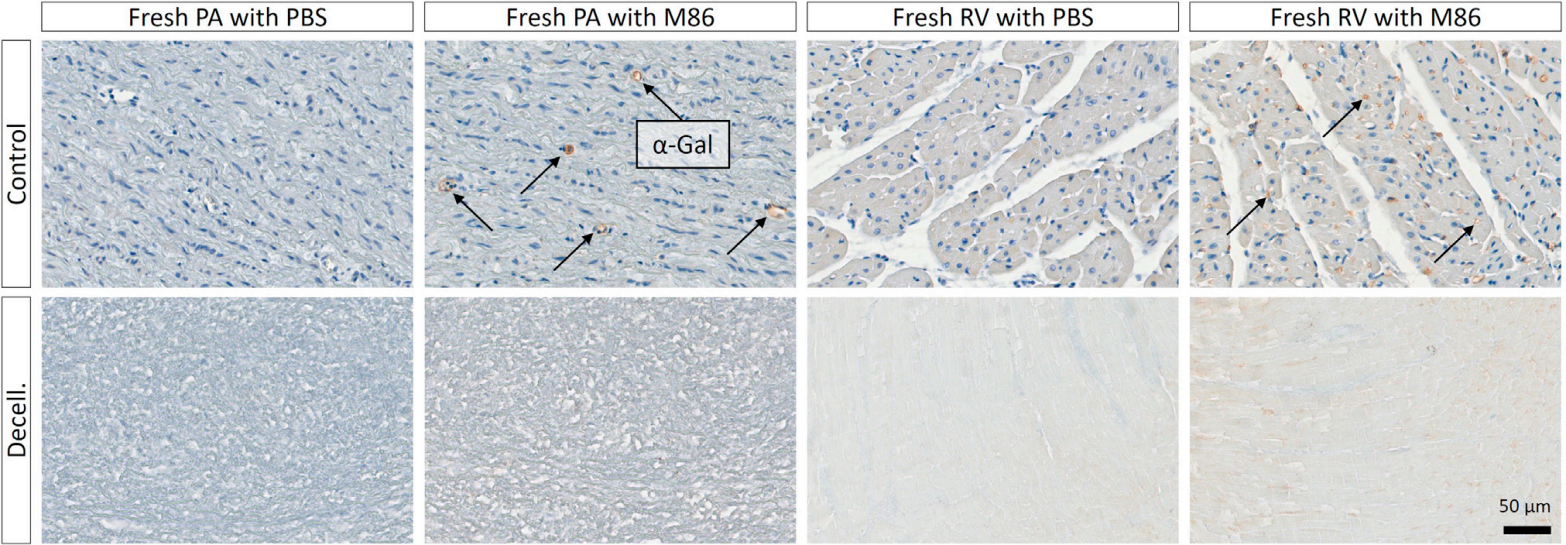
Representative results from the immunohistochemistry for the Galα1-3Galβ1-4GlcNAc-R (α-Gal) epitope in control and decellularized pulmonary artery (PA) and right ventricle (RV) cross sections counterstained with hematoxylin Incubation with PBS rather than M86 provides a reference point for antibody specificity and background staining. Punctate brown circles indicate positive α-Gal staining, marked by the black arrows. Decellularized (decell.) pulmonary artery and right ventricle demonstrate the absence of positive α-Gal staining. Native pulmonary valve leaflets were negative for α-Gal staining (data not shown). Fresh PA, n = 3; fresh RV, n = 3, fresh dPA, n = 3, fresh dRV, n = 3. Scale bar = 50 μm.

**Figure 11:**
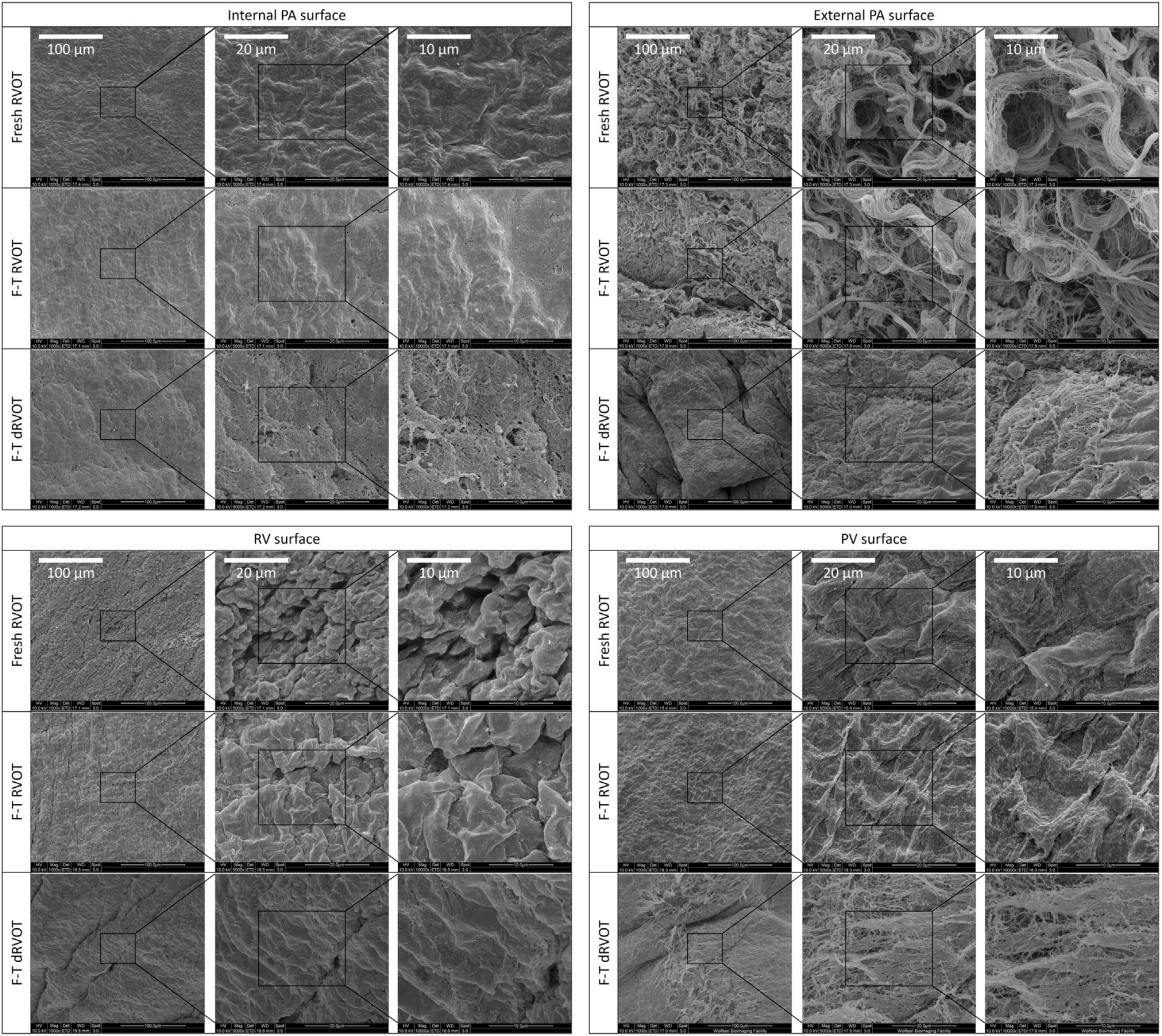
Representative results from scanning electron microscopy of fresh RVOT versus freeze-thawed (F-T) RVOT versus F-T and decellularized (d)RVOT surface topologies from the pulmonary artery (PA), right ventricle (RV), and pulmonary valve (PV) leaflet surfaces at increasing magnifications The native internal pulmonary artery surface is covered with a uniform endothelial cell layer resembling cobblestones, which becomes pitted in appearance upon freezing-thawing, indicating the start of mechanical cell destruction. The decellularized pulmonary artery internal luminal surface lacks this typical cobblestone morphology, with the cells having been removed and the underlying fibrous matrix uncovered. The external pulmonary artery surface is covered by an adventitial layer of collagen fibers, which are retained after the freeze-thawing process but removed by decellularization. Importantly, the underlying cells beneath the adventitia are also disrupted and are shown to be lifting off the underlying extracellular matrix. The native right ventricle surface has a pronounced confluent cell layer, which becomes pitted upon freeze-thawing the tissue. This monolayer is disrupted upon decellularization, with the underlying matrix becoming exposed and the cells removed. The pulmonary valve leaflet surface similarly shows a uniform cobblestone appearance in its native state, resembling that of the pulmonary artery, which is damaged upon freeze-thawing and completely removed following decellularization. Fresh dRVOT images are not shown for clarity. Fresh RVOT, n = 3; F-T RVOT, n = 3; F-T dRVOT, n = 3. Scale bars = 100 μm, 20 μm, or 10 μm, as indicated.

**Figure 12:**
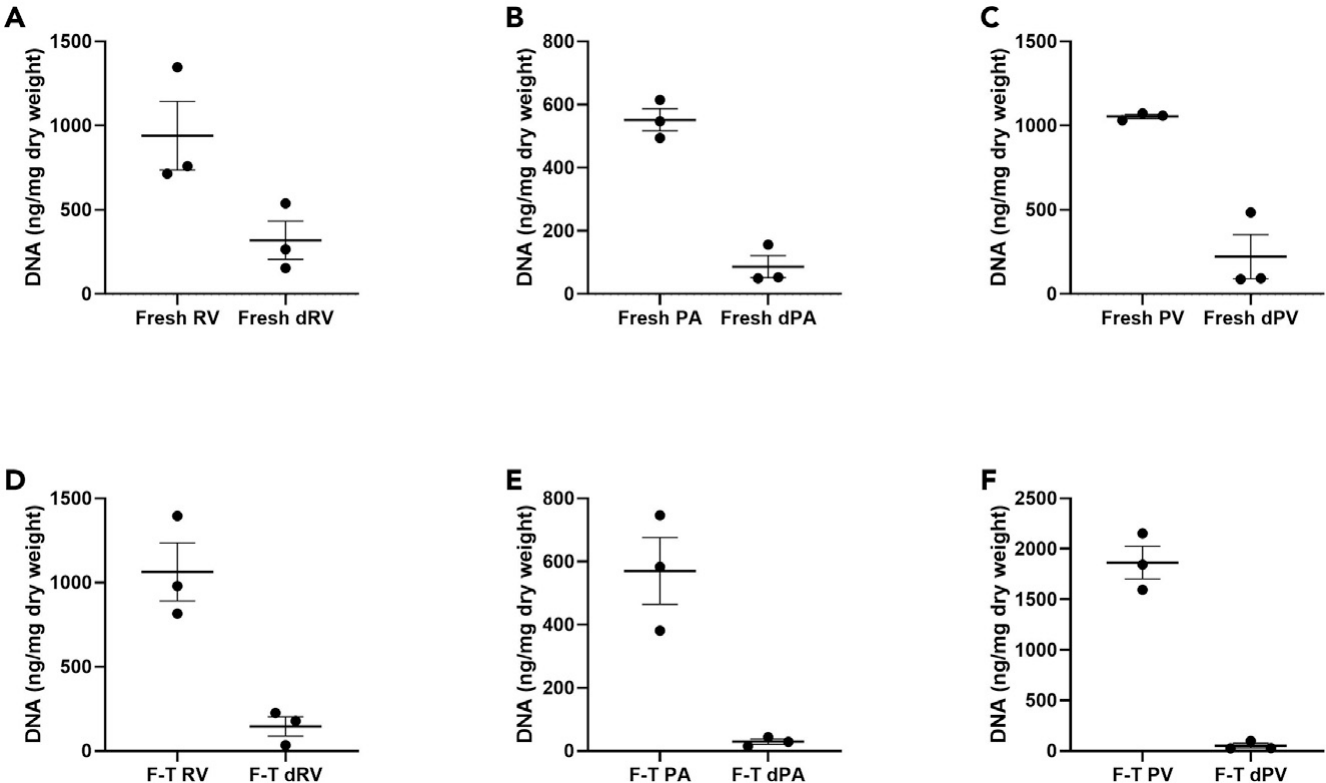
DNA quantification (A) Fresh right ventricle (RV) versus fresh decellularized right ventricle (dRV). (B) Fresh pulmonary artery (PA) versus fresh decellularized pulmonary artery (dPA). (C) Fresh pulmonary valve (PV) leaflet versus fresh decellularized pulmonary valve (dPV) leaflet. (D) Freeze-thawed right ventricle (F-T RV) versus freeze-thawed decellularized right ventricle (F-T dRV). (E) Freeze-thawed pulmonary artery (F-T PA) versus freeze-thawed decellularized pulmonary artery (F-T dPA). (F) Freeze-thawed pulmonary valve (F-T PV) leaflet versus freeze-thawed decellularized pulmonary valve (F-T dPV) leaflet. Graphs show the mean ± SEM from the depicted tissue type. Fresh RVOT, n = 3; fresh dRVOT, n = 3; F-T RVOT, n = 3; F-T dRVOT, n = 3.

**Figure 13:**
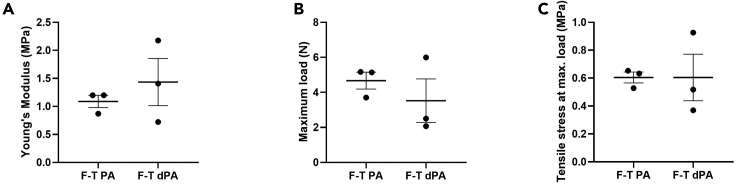
Representative data from axial mechanical testing of freeze-thawed pulmonary artery (F-T PA) versus F-T decellularized pulmonary artery (F-T dPA) (A) Young’s Modulus, (B) maximum load, and (C) tensile stress at maximum load is reported. Graphs show the mean ± SEM. F-T PA, n = 3; F-T dPA, n = 3.

This protocol decellularizes porcine RVOTs, removing immunogenic antigens and DNA whilst retaining the mechanical properties of the ECM. Histological/IHC analysis allows interrogation of the cross-sectional tissue structure, evaluating the removal of nuclei (H&E; Figure 8, DAPI; Figures 8 and 9) and immunogenic epitopes (IHC; Figure 10), and gross maintenance of the ECM (EVG; Figure 8). A clear reduction in the number of nuclei is observed in all tissue types (Figure 9), quantified from the DAPI staining. SEM provides information on the surface topology, and further confirms cell removal to reveal the underlying matrix of all tissue architectures (Figure 11). The histological readout from H&E and DAPI is complemented by DNA quantification, which provides evidence for a reduction in DNA in all tissue types (Figure 12). Further to this, tensile testing quantitatively validates the qualitative EVG results, with no difference in Young’s modulus, maximum load, or tensile stress at maximum load, indicating preservation of the scaffold’s mechanical properties following decellularization (Figure 13).

## Quantification and statistical analysis

Both steps for the nuclei quantification from DAPI images are carried out using ImageJ and use the ModularImageAnalysis (MIA) workflow automation plugin for ImageJ.^3^^,^^4^^,^^5^ These workflows are available at GitHub: Nuclei counting. The steps are summarized below.

Step 1: selection of regions of interest (ROIs).

1. A low resolution (downsampled) copy of the DAPI image is loaded.
2. Manually select five representative ROIs to process.
3. Regions are stored as ImageJ ROI files.

Step 2: counting nuclei within ROIs.

4. The high resolution region of the DAPI image is automatically loaded from the pre-selected ROI files.
5. Background subtraction and correction of the uneven illumination profile is achieved by dividing the DAPI image by the Gaussian-filtered copy (sigma = 65 μm).
6. Further background subtraction is carried out using the rolling ball filter (radius = 30 px).
7. The filtered image is binarized with a user-defined intensity threshold.
8. Holes smaller than 0.5 μm^2^ are removed from the binarized regions.
9. A median filter (radius = 0.3 μm) is applied to the binary image to smooth region edges.
10. Adjacent nuclei which have become merged during the binarization process are split using an intensity-based watershed operation.^6^
11. Nuclei are detected from the binarized image using connected components labeling, which identifies nuclei as contiguous regions of foreground-labeled pixels.^6^
12. Nuclei smaller than a user-defined area threshold are removed.
13. The number of nuclei per selected ROI as well as the statistics on nuclei areas are reported.
14. Average the nuclei count for each sample over the five distinct ROIs and quantify as nuclei/μm^2^.

***Note:*** For decellularized right ventricle samples, a manual counting method was necessary due to autofluorescence. This was achieved by taking the average of three independent manual counts of the nuclei in each ROI. The nuclei count for each sample was then averaged over the five distinct ROIs and quantified as nuclei/μm^2^.

For DNA quantification, Excel is used to process the data. Table 1 shows a worked example to process the raw data as described below.

15. Calculate the average and standard deviation of the 8 blanks.

**CRITICAL:** If the standard deviation is ≥ 10% of the mean, the absorbance values at the lowest end of the curve will be inaccurate due to baseline absorbance variability across the plate. Step 72. Elimination of single stranded nucleic acid contaminants and step 73. Quantification of double stranded DNA should be repeated.

16. Plot the corrected standard curve, normalized against the average of the blanks.
17. Calculate R^2^.

***Note:*** An R^2^ of ≥0.98 was used as the criteria to include or exclude the experiment.

**CRITICAL:** If the R^2^ is < 0.98, repeat step 72. Elimination of single stranded nucleic acid contaminants and step 73. Quantification of double stranded DNA.

18. Calculate the concentration of DNA in the well using the equation of the line with the unknown sample corrected absorbance (absorbance value less the average of the blanks).
19. Account for the dilution factor to calculate the true concentration of the sample.
20. Normalize concentration to dry weight to calculate the ng/mg tissue.

## Limitations

The potential risk of microbial contamination associated with using non-sterilized cardiac tissue is a drawback of this protocol, particularly when working at 37°C–42°C, an optimal environment for infection. However, we have observed that proper handling in combination with the inclusion of P/S in all solutions greatly decreases this risk. Limited tissue mass, particularly regarding the pulmonary valve leaflets, poses a challenge to full characterization. When working with small tissue quantities, characterization must be carried out within the limits of feasibility. The mechanical testing, for example, uses significantly more pulmonary artery than histological analysis, SEM, and DNA isolation, combined. While histology provides a robust and reproducible outcome, to quantitatively evidence the absence of DNA requires a highly sensitive assay. Traditional nanospectrophotometers are not appropriate for such applications when specifically interrogating the presence of double stranded nucleic acids. Even when using a plate reader, reading values at the detection limit of the equipment means all data must be considered within the degree of variance across the plate. Lastly, the inherent variability when working with primary tissue is unavoidable, and as such we recommend assessing all data in a minimum of triplicate repeats.

## Troubleshooting

### Problem 1

Sourcing porcine RVOTs (related to part 2: preparation of tissue: dissecting, washing, and freezing porcine RVOTs).

### Potential solution

Sourcing porcine RVOTs as a byproduct of the food industry can be an alternative to sourcing from an animal research unit. In our experience, sourcing from an abattoir has resulted in a higher proportion of unsuitable tissue due to damage incurred during explant. Particular care must be taken to ensure RVOTs are undamaged before use.

### Problem 2

Leakage from hose barb connectors/taps or the chamber (related to part 3c: assemble the decellularization apparatus).

### Potential solution

**Figure 14:**
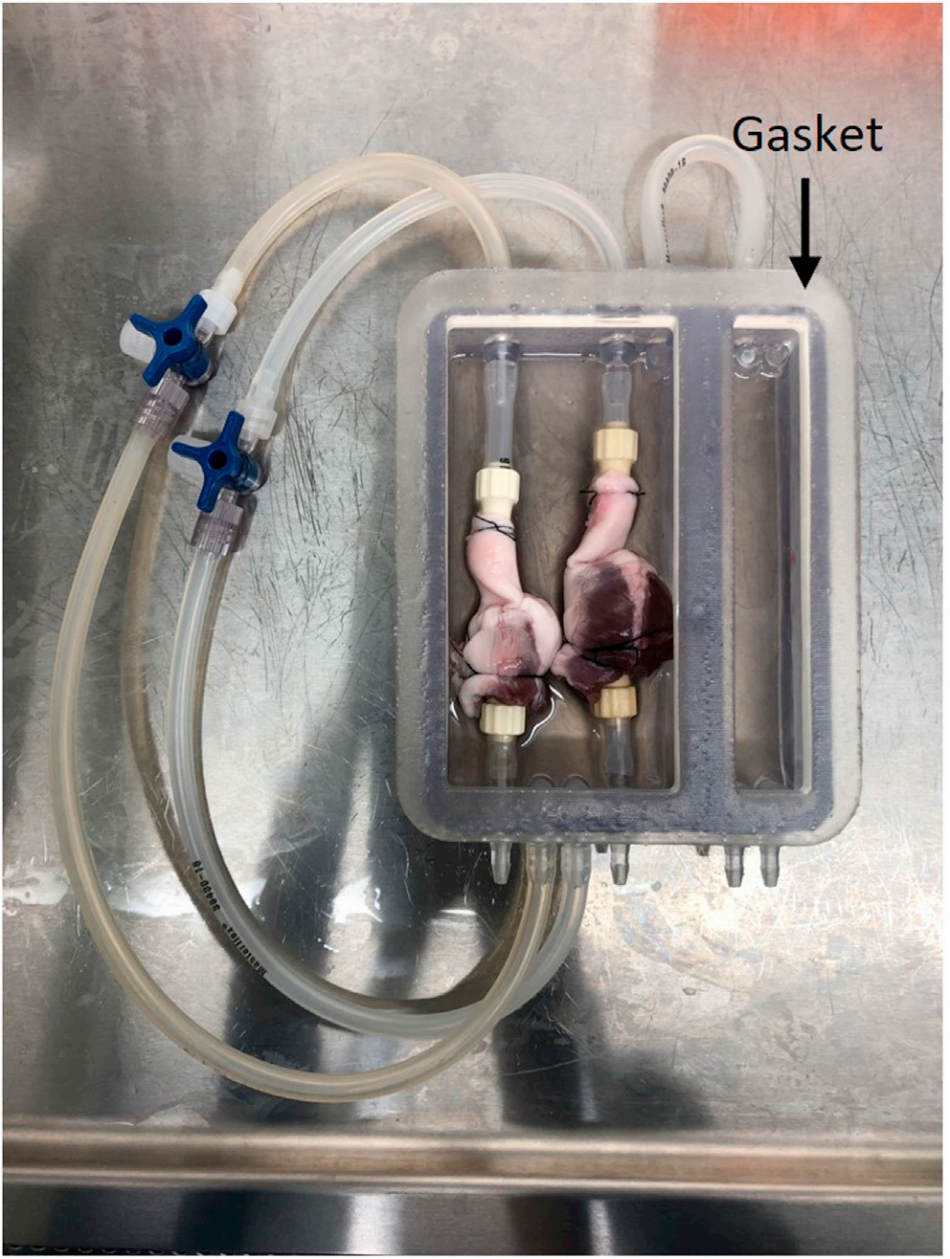
3D printed flow chamber with two mounted RVOTs and the gasket in place (black arrow)

First, visually assess the source of leakage. Leakage from hose barb connectors/taps will be due to insufficient tightening and can be easily resolved by securing the loose connection. Leakage from the chamber itself is most likely due to an improper seal between the chamber, gasket, and lid. If leakage occurs from the chamber, initially assess whether the clamps are suitably tightened. Having checked the clamps and established they are secure, evaluate the placement of the gasket, which should cover the chamber footprint (Figure 14).

If the gasket is improperly placed, remove the solution with a 50 mL sterile syringe and place it in a sterile container. Next, disassemble the tubing from the chamber and move the chamber to the class II microbiological safety cabinet. Remove the clamps and take off the lid. Adjust the gasket and reseal the chamber. Once the chamber is resealed and back in the incubator with the relevant tubing attached, place the solution back into the chamber and reassess leakage.

### Problem 3

The tissue comes loose under flow, dislodging from the barb (related to part 3d: decellularization procedure). This will prevent flow through the lumen of the RVOT and reduce decellularization success.

### Potential solution

Remove the solution with a 50 mL sterile syringe and place it in a sterile container. Next, disassemble the tubing from the chamber and move the chamber to the class II microbiological safety cabinet. Remove the clamps and take off the lid and gasket, before removing the RVOT and relevant hose barbs.

The most likely cause of the RVOT detaching from the hose barbs is loose sutures. Remount the RVOT, as described in detail in part 3b: prepare and mount the RVOT. Another possible reason for tissue detachment is a blockage in the lumen of the RVOT, which could be caused by incomplete removal of debris during the washing process.

Once the chamber is resealed and back in the incubator with the relevant tubing attached, place the solution back into the chamber and continue the protocol. Always depress the syringe slowly and do not apply excessive pressure to reduce the risk of tissue coming loose. If filling causes pressure buildup, the filter may be blocked. Replace this filter regularly.

### Problem 4

Microbial contamination (related to part 2: preparation of tissue: dissecting, washing, and freezing porcine RVOTs and part 3: decellularization of porcine RVOTs). Although precautions are taken to reduce microbial contamination, the risk cannot be eliminated. There are two primary reasons for microbial contamination. The first is contamination originating from the tissue itself, present from the start of the process due to the unsterile harvesting conditions. The second is user-derived contamination during washing and/or decellularization processes.

### Potential solution

Contamination from the tissue, present from the beginning of the procedure.

• Thoroughly inspect the tissue for warnings of contamination following dissection (part 2: preparation of tissue: dissecting, washing, and freezing porcine RVOTs) and discard those with signs of infection. Where there is doubt, consider discarding the RVOT, particularly as the dual chamber system means contamination present in one sample will cause contamination of the other.
• Increase the antibiotic concentration in the solutions. P/S is used at a working concentration of 1×; however, it is possible to increase this to 5× if contamination is a recurrent problem.

Contamination during ongoing washing and/or decellularization.

**Figure 15:**
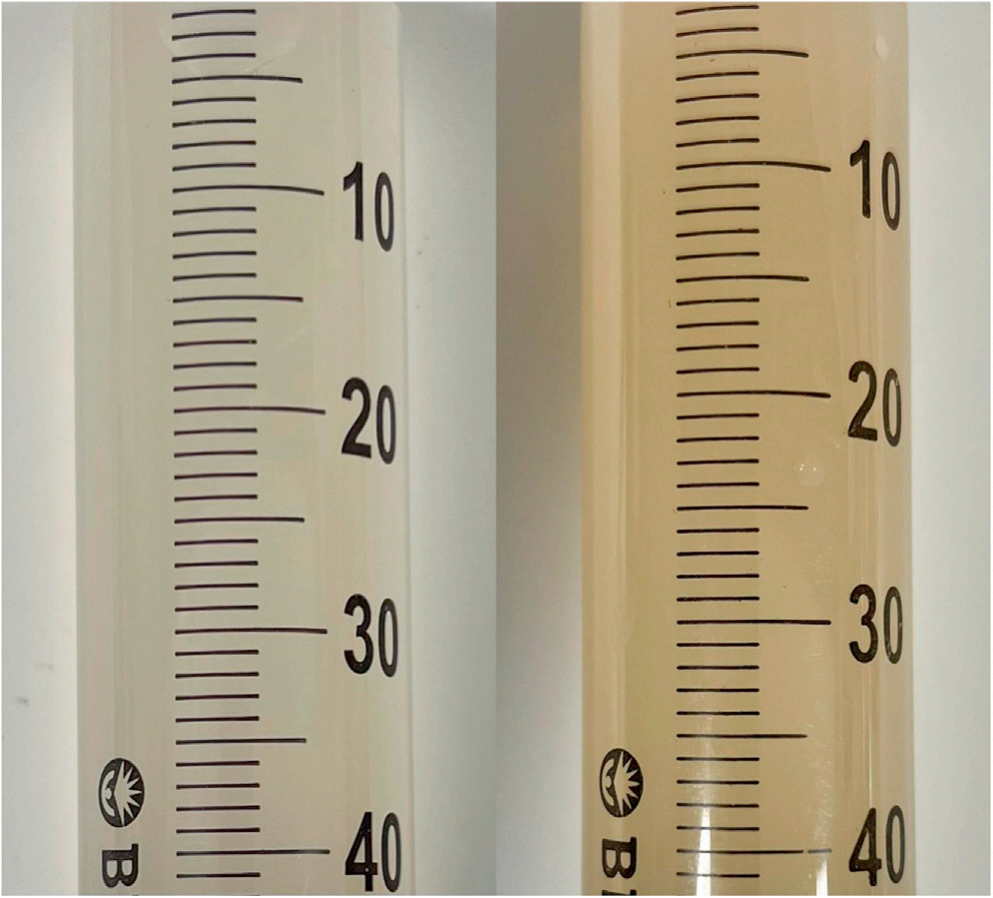
Examples of contaminated decellularization solutions

• Ensure all forceps, scissors, glassware, and containers are sterile before use. If not autoclavable, the use of 70% (v/v) ethanol to thoroughly sterilize apparatus is appropriate.
• Thoroughly and regularly clean the incubator and microbiological safety cabinet with 70% (v/v) ethanol.
• Always autoclave or filter sterilize solutions.
• Do not place forceps, scissors, or tissue directly on the working area of the bench or microbiological safety cabinet, instead use a sterile surface.
• Change the filter on the chamber regularly.
• Regularly check for signs of microbial contamination by visual inspection of the solution. Macroscopic signs of contamination include cloudy solution and/or an unpleasant odor. Signs of contamination may be more subtle, but will become apparent at later stages if present, particularly after circulating the same solution for ∼63–66 h (days 5–6). Examples of contaminated solutions are shown in Figure 15.

In cases where no nuclei are seen in the histological stains, but the DNA quantification indicates the presence of nucleic acids, microbial contamination could be the cause of the seemingly conflicting results. Infection must reach a certain detection limit before becoming apparent and if bacteria are present below this threshold, the contamination could be undetected. The quantification of dsDNA does not distinguish microbial from porcine DNA; thus, it is possible to find high levels of DNA even in the absence of nuclear staining.

### Problem 5

Incomplete decellularization (related to part 4: characterization of decellularized tissue). The most efficient way to initially assess whether cells have been removed is to carry out histological staining of tissue cross sections with H&E and DAPI. If nuclei are evident, decellularization is incomplete.

### Potential solution

If H&E and DAPI demonstrate incomplete removal of nuclei, poor flow through the chamber may be the cause. The continuous mechanical decellularization provided by the flow system has two key functions: firstly, flow enhances the effectiveness of decellularization, and secondly, flow allows the decellularization solutions to contact the internal RVOT surface, including the pulmonary valve, essential when working with a 3D structure.

• Flow from the sample chamber to the reservoir chamber (purple arrow, Figure 4) should be apparent when the pump is switched on.
• In order to regularly check the there are no blockages in any tubes of the system, observe solution flowing through all tubes when filling via both primary taps.
• If filling causes pressure buildup, the filter may be blocked. Replace this regularly.

## Resource availability

### Lead contact

Further information and requests for resources and reagents should be directed to and will be fulfilled by the lead contact, Francesca Bartoli-Leonard (f.bartoli-leonard@bristol.ac.uk).

### Technical contact

Further information requests on the technical specifics of performing this protocol should be directed to and will be fulfilled by the technical contact, Amy G. Harris (a.harris@bristol.ac.uk).

### Materials availability

This study did not generate new unique reagents.

### Data and code availability

The 3D printing .stl files generated during this study have been deposited at GitHub: 3D flow rig for decellularization, https://doi.org/10.5281/zenodo.10520001. Workflows for nuclei counting have been deposited at GitHub: Nuclei counting, https://doi.org/10.5281/zenodo.10520008. All original data are available from the corresponding author upon request.
